# The roles and targeting options of TRIM family proteins in tumor

**DOI:** 10.3389/fphar.2022.999380

**Published:** 2022-09-30

**Authors:** Yuxin Zhang, Wenzhou Zhang, Lufeng Zheng, Qianqian Guo

**Affiliations:** ^1^ Department of Pharmacy, The Affiliated Cancer Hospital of Zhengzhou University and Henan Cancer Hospital, Zhengzhou, China; ^2^ School of Life Science and Technology, Jiangsu Key Laboratory of Carcinogenesis and Intervention, China Pharmaceutical University, Nanjing, China

**Keywords:** TRIM family proteins, tumor, targeted therapy, post-translational modification, PROTAC

## Abstract

Tripartite motif (TRIM) containing proteins are a class of E3 ubiquitin ligases, which are critically implicated in the occurrence and development of tumors. They can function through regulating various aspects of tumors, such as tumor proliferation, metastasis, apoptosis and the development of drug resistance during tumor therapy. Some members of TRIM family proteins can mediate protein ubiquitination and chromosome translocation *via* modulating several signaling pathways, like p53, NF-κB, AKT, MAPK, Wnt/β-catenin and other molecular regulatory mechanisms. The multi-domain nature/multi-functional biological role of TRIMs implies that blocking just one function or one domain might not be sufficient to obtain the desired therapeutic outcome, therefore, a detailed and systematic understanding of the biological functions of the individual domains of TRIMs is required. This review mainly described their roles and underlying mechanisms in tumorigenesis and progression, and it might shade light on a potential targeting strategy for TRIMs in tumor treatment, especially using PROTACs.

## 1 Introduction

In 2020, there were an estimated 19.3 million new cancer cases and nearly 10 million cancer deaths worldwide, and by 2040, global cancer is projected to reach 28.4 million cases, a 47% increase from 2020 (Sung et al., 2021). Malignant tumor has become a worldwide disease threatening human health due to its increasing incidence. Multiple factors and signal transductions are complicated in tumor progression, and the underlying mechanisms have been widely explored. Currently, the treatment methods of malignant tumors are mainly through surgery, radiotherapy, chemotherapy, targeted therapy, and immunotherapy (Gotwals et al., 2017; Stine et al., 2022). However, current therapeutic methods are still unable to completely eradicate tumors, which might be due to the fragmentary mechanisms underlying tumor progression (Wang et al., 2018; Mullard, 2020). Additionally, although many oncogenes (such as *HER2, K-ras, EGFR*) and tumor suppressor genes (such as *p53, PTEN, Rb*) have been found and several targeted-drugs (such as targeting *EGFR, HER2*) have been successfully applied to the clinical treatment, drug resistance often occurs during treatment. Thus, it may overcome the drug resistance and facilitate the drug development by identifying new drug targets (De Luca et al., 2008; Oh and Bang, 2020).

Protein post-translational modification (PTM) is critical for tumor progression, based on which some methods or targets have been explored for anti-tumor drug development, such as the Proteolysis-Targeting Chimeras (PROTAC) technology. Protein ubiquitination is an ubiquitous form of PTM, which occurs through a three enzyme cascade [Ubiquitin-activating Enzyme 1 (E1), Ubiquitin-activating Enzyme 2 (E2), Ubiquitin Ligase 3 (E3)]. Studies have confirmed that protein ubiquitination is closely related to the occurrence and development of tumors (Song and Luo, 2019). On the basis of their catalytic mechanism, E3 ubiquitin ligases are divided into HECT domain family, RING domain family, and RING1-in-between-RING2 (RBR) type U-box protein family (Dale et al., 2021). They are mainly responsible for connecting target proteins with specific E2 ubiquitin ligases, determining the specific recognition of target proteins, and playing an important role in the ubiquitination pathway (Zhao et al., 2021).

Tripartite motif (TRIM) family proteins are one of the sub-families of the RING-type E3 ubiquitin ligases, which comprises cyclic lipid domains, and more than eighty TRIM proteins exist in human beings. Some members of TRIM family proteins play an important role in tumor occurrence and development, for example, TRIM8, TRIM24, TRIM28, and TRIM32 are found to be highly expressed in malignant tumor tissues and participate in the malignant biological behavior (Cambiaghi et al., 2012; Mohammadi et al., 2022). This paper summarized the effects and underlying mechanisms of TRIMs on tumorigenesis and development, so as to provide a new direction for the targeting therapy of malignant tumors.

## 2 Materials and methods

### 2.1 Search strategy and inclusion and exclusion criteria

The NCBI PubMed database was searched and all eligible articles published before 20 August 2022 were selected. Keywords and search terms included “TRIM” or “TRIM Protein” or “TRIM Protein family” or “TRIM and tumor” or “TRIM and cancer” or “TRIM and Tumor Proliferation” or “TRIMs and Tumor metastasis” or “TRIMs and Chemoresistance” or “TRIMs and p53″ or “TRIMs and NF-κB” or “TRIM and Autophagy.” Use these terms to find the most suitable references possible. In addition, the references in this article are manually collated by Yuxin Zhang and Wenzhou Zhang. The language of the retrieved studies was limited to English.

Eligible for the study:1) full text; 2) English studies; 3) Research on TRIM family and tumor; 4) The relationship between TRIM protein expression and clinicopathological parameters or tumor survival. The exclusion criteria were as follows:1) studies not in English; 2) non-human research; 3) Reviews, reviews, case reports, letters, and meta-analyses; 4) Research on diseases other than cancer; 5) Non-TRIM pedigree study; 6) The study lacked useful information, such as clinical features and survival curves. All evaluations were conducted independently by four researchers to ensure accurate pre-proofreading of articles by the journal. All authors discuss any conflicts to reach a consensus.

The following information was extracted from each included study: basic information including first author name, year of publication, country, name of TRIM protein, cancer type, related mechanism of TRIM action, expression and effect of TRIM protein in tumors, and detection method.

The flow chart of literature selection is shown in Supplementary Figure S1.

## 3 Overview of TRIMs

### 3.1 Structure of TRIMs

TRIMs exist in all multicellular animals and more than eighty kinds of TRIMs have been identified in human (Mohammadi et al., 2022). TRIMs are characterized by an N-terminal construction region which contains three typical domains, namely RING-finger domain, one or two b-box domains, and coil-coil region (Crawford et al., 2018).

The RING finger domain generally starts from 10–20 amino acid residues at the N-terminal region and is formed by the arrangement of cysteine and histidine. There is a pair of cysteine residues and histidine residues at the N-terminal region and C-terminal region, respectively, these four residues can form a cave to combine two zinc atoms (Ikeda and Inoue, 2012). The binding with zinc atom can mediate the ubiquitination reaction of protein itself or different substrates. Therefore, RING finger domain has become the symbol of many E3 ubiquitin ligases (Joazeiro and Weissman, 2000). Most TRIMs, such as TRIM47, TRIM24, TRIM50, TRIM59, are defined as E3 ubiquitin ligases because of their zinc finger domain. However, not all TRIMs have zinc finger domains, such as TRIM14, TRIM16, and TRIM20, which are considered not to have E3 ubiquitin ligase activity, these TRIMs without zinc finger domain also exist in human body (Zhao et al., 2021).

B-box domain is also a zinc ion binding domain, which is divided into B-Box 1 and B-Box 2. The difference between these two types is that the second binding site of B-Box 1 is cysteine and that of B-Box 2 is histidine. The function and role of B-box domain are still fragmentary (Zhan and Zhang, 2021). However, they have been shown to be critical for TRIM activity, the B-Box1 structure closely resembles the folds of the RING, ZZ and U-box domains of E3 and E4 ubiquitin enzymes, raising the possibility that the B-box1 domain either has E3 activity itself or enhances the activity of RING type E3 ligases (Massiah et al., 2006). For example, TRIM29 belongs to the acyclic group of TRIM protein family members, which consists of a b-box domain and four zinc-finger motifs in a coiled-coil domain, and uses the B-box domain as an E3 ubiquitin ligase in place of RING (Hsu et al., 2021). The B-box can occupy an E2 binding site on the catalytic RING domain by mimicking E2-E3 interactions, inhibiting TRIM21 ubiquitination (Dickson et al., 2018). Furthermore, B-box interfaces not only impair oligomerization *in vitro* but also abolish ProMyelocyticLeukemia (PML) sumoylation and nuclear body biogenesis (Li Y. et al., 2019).

The coiled-coil domain is well-conserved across all TRIM proteins and often located after B-Box2. The length of gene encoded by this domain is about 100 bases. This domain mainly participates in the interaction of homodimers, the formation of macromolecular complexes, and determining the subcellular localization of proteins (Reymond et al., 2001; Zhan and Zhang, 2021). For example, the interaction between the breast cancer Susceptibility 1 (BRCA1) protein and the DNA damage repair protein (PALB2) is mediated by associations in predicted helical coiled-coil regions, and these two proteins mediate the homodimer of PALB2 through antiparallel coiled-coil interactions (Song et al., 2018); The four zinc-finger motifs in the coiled-coil domain of TRIM29 are also involved in the formation of homodimers and heterodimers associated with DNA binding (Hsu et al., 2021). Notably, the necessity of the coiled-coil domain is highlighted by the results that this domain can be bound by viral proteins, thus preventing viral protein activation, substrate recruitment and/or imparting allosteric changes (Weinert et al., 2015; Koliopoulos et al., 2018).

**FIGURE 1:**
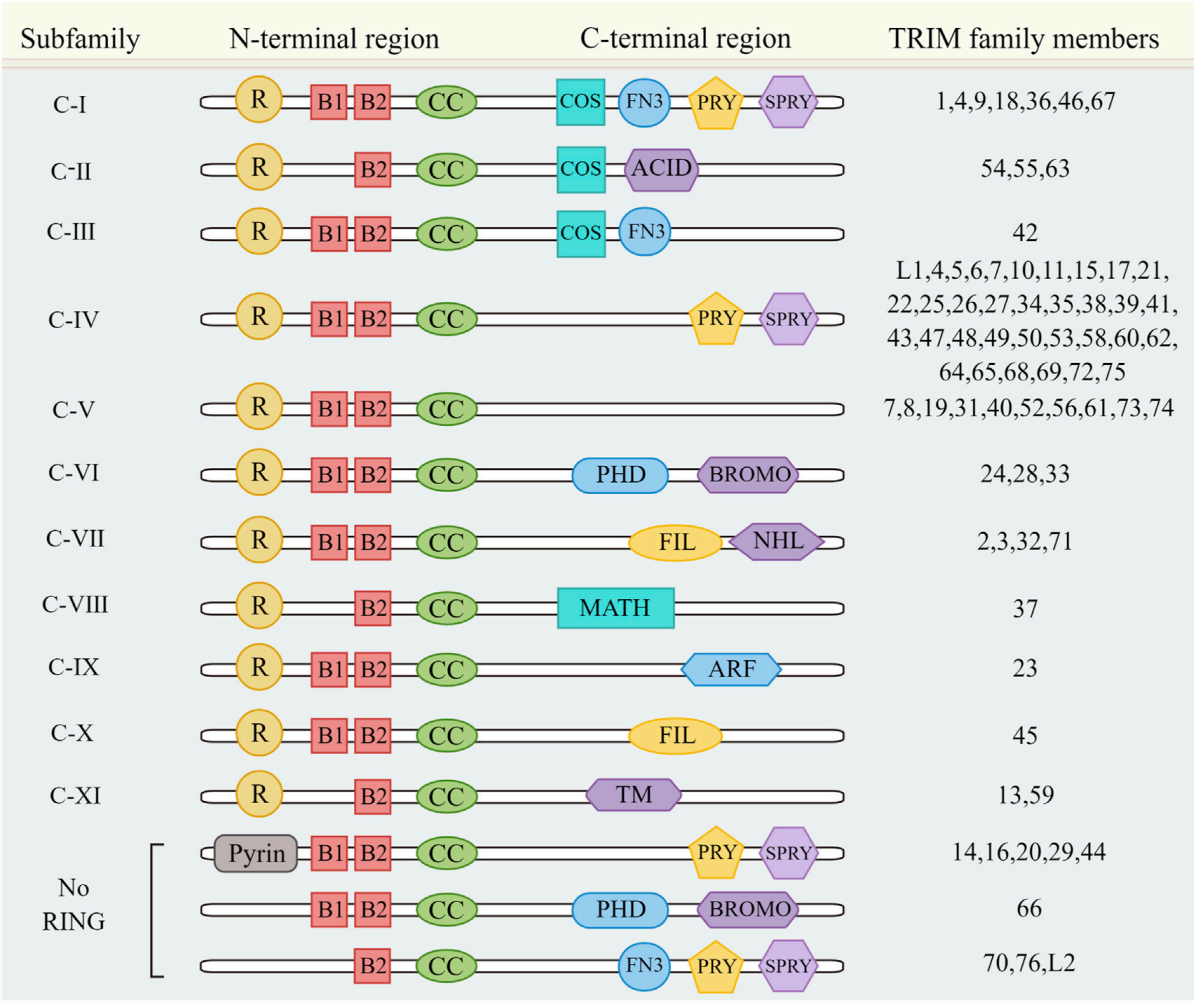
TRIM family protein classification. TRIM family proteins were divided into 11 subfamilies according to the different C-terminal domains. Figure 1 also includes a special case of a TRIM family member without a RING domain.

In addition, TRIM family also has a complex C-terminal domain, including COS domain, SPRY-associated (PRY) domain, SPIa and the ryanodine receptor (SPRY) domain, acid rich region (ACID), serine IG domain (FIL), NHL domain, bromine domain (BROMO), Meprin and TRAF homologous domain (MATH), ADP ribosylation factor family domain (ARF), and transmembrane region (TM). The PRY-SPRY domain plays an important role in innate immune responses, recognition of viral proteins, and subcellular localization of certain TRIM proteins (James et al., 2007), such as the RNA-binding activity of TRIM25 is mediated by its PRY/SPRY domain, which is also necessary for TRIM25 ubiquitination (Choudhury et al., 2017); The PRY-SPRY domain of TRIM14 mediates protein interactions and interacts with different partners to exert individual functions (Yu et al., 2019). Based on the different C-terminal domains, TRIM family proteins can be divided into 11 subfamilies from Ⅰ to XI (Figure 1) (Hatakeyama, 2017).

### 3.2 Functions of TRIMs

TRIMs are involved in various physiological processes, including cell proliferation, DNA damage repair, intracellular signal transduction, and the immune response. Meanwhile, TRIM29 is also engaged in the occurrence and development of different diseases, including cancer, inflammatory diseases, infectious diseases, neuropsychiatric diseases, chromosome abnormalities, and developmental diseases (Wan et al., 2021; Meroni and Desagher, 2022). TRIM29 is one of the components of the DNA repair complex and can act as a scaffold protein to assemble DNA repair proteins into chromatin, and then efficiently promote DNA repair during DNA damage repair (Masuda et al., 2015). TRIMs also engage in host antiviral infection; for example, TRIM23 plays an antiviral role in herpes simplex virus 1 (HSV-1), cerebral myocarditis virus (EMCV) and influenza A virus (IAV) by activating TBK1 to mediate virus-induced autophagy (Sparrer et al., 2017); TRIM35 participates in RIG-I antiviral immunity and IAV infection defense mechanisms by catalyzing Lys63-or Lys48-linked polyubiquitin, mediating protection against influenza infection (Sun et al., 2020); TRIM14 interacts with the mitochondrial junction molecule MAVS (mitochondria antiviral signaling) in the RLRs (RIG-I like receptors) signaling pathway through its pry-spry domain and interacts with the natural immune regulator NEMO (NF-κB essential modulator) protein, thus regulating host innate immune response caused by the virus (Zhou et al., 2014; Yu et al., 2019). TRIM5α, as a restriction factor, can recognize the capsid structure of HIV-1, prevent the reverse transcription of the virus genome, inhibit the nuclear transfer of HIV-1 virus DNA, and induce the rapid degradation of viral Gag protein to block the release of virus particles, indicating that TRIM5α can specifically inhibit HIV-1 infection by suppressing the replication of HIV-1 virus in several ways (Luban, 2012; Nakayama and Shioda, 2015; Ganser-Pornillos and Pornillos, 2019). Similarly, TRIM11 can bind to HIV-1 capsid protein and inhibit the reverse transcription of viral protein (Yuan et al., 2016); TRIM22 prevents the transfer of viral structural proteins and inhibits the replication of HIV-1 through its E3 ubiquitin ligase activity (Turrini et al., 2015; Vicenzi and Poli, 2018); TRIM37 can directly enter virus particles and suppress the synthesis of virus DNA (Tabah et al., 2014); TRIM28 limits HIV-1 infection by inhibiting virus integration into the host genome (Ma et al., 2019). These TRIMs can be modulated to defend against HIV infection to some extent, and hopefully become novel suppressors for AIDS treatment.

Additionally, TRIMs are involved in regulating neurodegenerative diseases. TRIM32 has anti-neuronal apoptosis activity and can be used as a new target candidate for treating Alzheimer’s disease (Ntim et al., 2020). TRIM6 and TRIM24 play an important role in the early stage of Parkinson’s disease and can be used as early warning genes for the development of Parkinson’s disease (Nenasheva et al., 2017; Li C. et al., 2021). Furthermore, the effects of TRIMs on tumorigenesis have also attracted increasing attention, with many studies have been conducted in recent years. Therefore, in this review, we further focused on summarizing the various aspects of TRIMs in tumorigenesis.

## 4 The functional mechanisms of TRIMs in tumorigenesis and progression

### 4.1 TRIMs and ubiquitination

Ubiquitination modification plays a critical role in cell cycle distribution, cell growth, apoptosis, DNA damage repair, and the immune response. It is mainly catalyzed by tertiary enzyme-linked enzymes, including E1 ubiquitin activating enzyme, E2 ubiquitin binding enzyme, and E3 ubiquitin ligase (Deng et al., 2020). Most TRIMs contain the N-terminal ring domain, which endows the E3 binding activity of this protein family and participates in the occurrence and development of tumors by regulating gene expression. Currently, numerous TRIMs have been found to have E3 ubiquitin ligase activity (Hatakeyama, 2017; Venuto and Merla, 2019).

TRIM6 facilitates the ubiquitination of tuberous sclerosis proteins TSC1 and TSC2, which are two negative regulators of the mTORC1 pathway, thus activating the mTORC1 pathway to induce renal fibrosis, which is tightly correlated with renal carcinoma occurrence (Liu W. et al., 2020). Similarly, TRIM37 is upregulated in renal cell carcinoma and promotes the occurrence and development of renal cell carcinoma by mediating histone H2A ubiquitination resulting in activation of TGF-β1 (Miao et al., 2021). TRIM15 interacts with APOA1 through its PRY/SPRY domain, and mediates the ubiquitination and degradation of APOA1 dependent on its RING domain, thereby promoting the invasion and metastasis of pancreatic cancer cells (Sun et al., 2021). In addition, TRIM28 can form complexes with MAGE and can mediate the ubiquitination and proteasome degradation of tumor suppressor proteins, such as 5′-adenosine monophosphate lipid-activated protein kinase (AMPK) and p53 (Pineda and Potts, 2015; Schafer et al., 2021). Another study also indicated that TRIM31 inhibits MDM2-mediated ubiquitination of p53 by competitively inhibiting the MDM2-p53 interaction, resulting in the stabilization and activation of p53. This process has a huge effect on the occurrence and development of breast cancer (Guo et al., 2021). TRIM47 increases p53 ubiquitination and degradation by interacting with p53 protein, thus promoting the malignant biological behavior of renal cell carcinoma (Table 1) (Supplementary Table S1) (Chen et al., 2021).

**TABLE 1:** Relationship between TRIMs and ubiquitination.

Name	Ubiquitination targets	Effects	References
TRIM6	Tuberous sclerosing protein (TSC) 1 and 2	Promote renal fibrosis, leading to progressive loss of renal function	Liu et al. (2020)
TRIM15	APOA1	Promote invasion and metastasis of pancreatic cancer cells	Sun et al. (2021)
TRIM28	AMPK	Promote the proliferation and metastasis of tumor cells	Pineda and Potts (2015)
	PRKAA1/AMPKα1	Autophagy is reduced and cellular metabolic changes, including upregulation of mTOR signaling	Schafer et al. (2021)
TRIM31	MDM2 and p53	Lead to the stabilization and activation of p53 and inhibits the proliferation, migration and invasion of breast cancer cells	Guo et al. (2021)
TRIM47	p53	Promoting the malignant biological behavior of renal cell carcinoma	Chen et al. (2021)
TRIM37	Histone H2A	Promote EMT and malignant progression of RCC cells	Miao et al. (2021)

### 4.2 TRIMs and chromosome translocation and transcription

**FIGURE 2:**
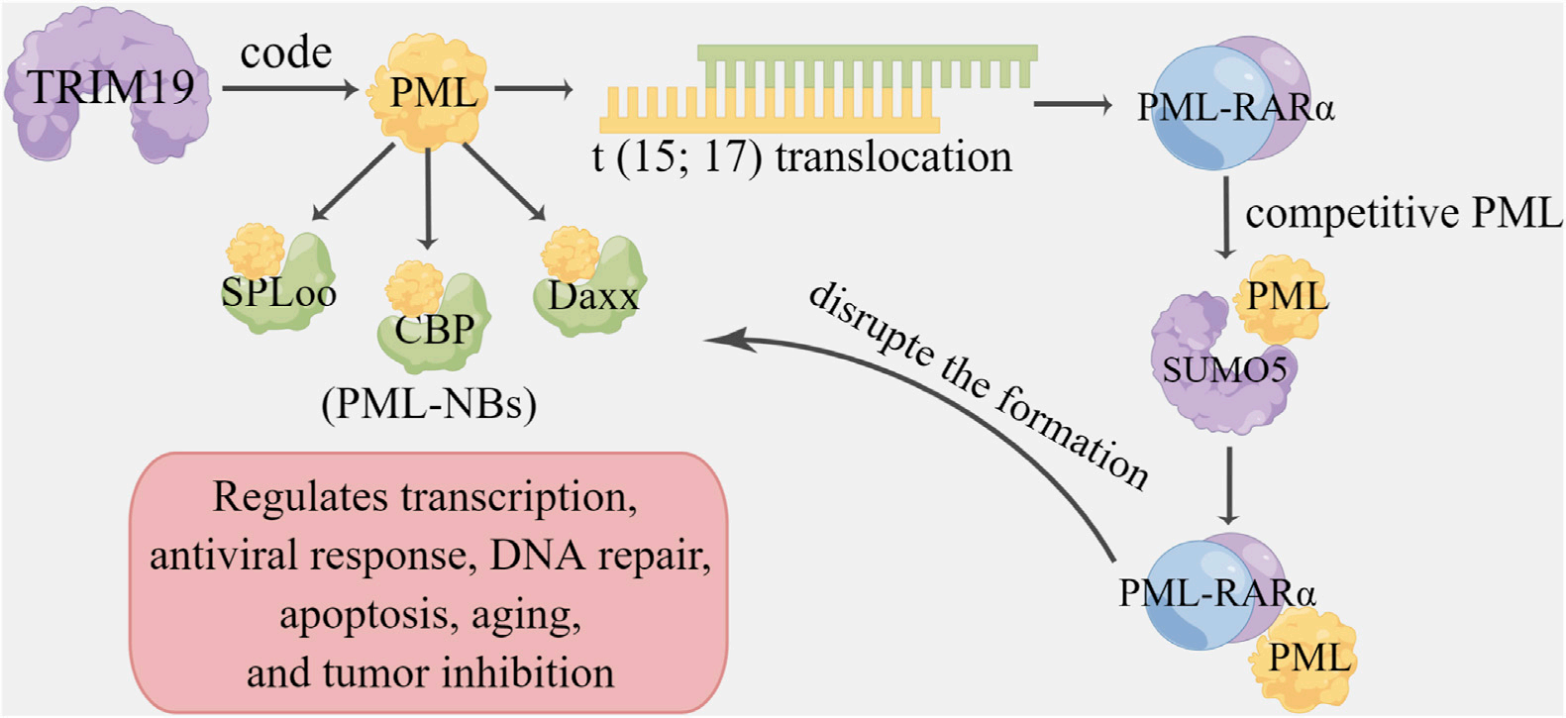
TRIMs and chromosome translocation and transcription. TRIM19 is involved in encoding promyelocytic leukemia gene PML, which is fused into PML-RARα and PML-NBS, and plays a role in tumor genesis and development. The promyelocytic leukemia gene (PML) encodes TRIM19, which is characterized by its involvement in translocation T (15; 17), resulting in the PML-retinoic acid receptor-α (RARα)) fusion protein. PML can interact with SPLoo, CBP and Daxx to form PML nuclear bodies (PML-NBSs), which regulate transcription, antiviral response, DNA repair, apoptosis and tumor suppression. PML-RARα can also competitively bind to PML *via* SUMO5, thereby disrupting PML-NBS formation.

Chromosomal translocation produces abnormal proteins, which can affect tumor progression mainly by regulating receptors, such as retinoic acid receptor *α* (RARα) and hormone receptors (Hsu and Kao, 2018). Some TRIM genes are often transferred to other genes to produce fusion proteins involved in cancer initiation and progression, the most famous of which is the promyelocytic leukemia gene *PML*, which encodes TRIM19 (Cambiaghi et al., 2012; Dutrieux et al., 2015). *PML* specifically participates in translocation occurring in acute promyelocytic leukemia (APL) to form the PML-retinoic acid receptor *α* (PML-RARα) fusion protein (Jensen et al., 2001; Mohammadi et al., 2022). This fusion protein, named PML-RARα, can inactivate the PML protein and interfere with the signal transduction of RARα to inhibit cell differentiation and promote cell proliferation (Liquori et al., 2020). PML-RARα can also competitively bind to PML with SUMO5, thus disrupting the formation of PML-NBS (Bernardi et al., 2004; Liang et al., 2016; Chang et al., 2018). Additionally, PML can also interact with SPLoo, CBP, and Daxx to form PML nuclear bodies (PML-NBs), which are nonmembrane binding domains in the nucleus that regulate transcription, the antiviral response, DNA repair, apoptosis, and tumor inhibition (Zhong et al., 2000; Lindsay et al., 2008; St-Germain et al., 2008). TRIM24, or TIFlα, which is a member of the TIFl family, participates in regulating gene transcription and is recognized as a coregulator of retinoic acid signaling (Khetchoumian et al., 2007). Furthermore, TRIM24 forms a carcinogenic fusion protein through chromosome translocation, subsequently promoting the occurrence and development of acute promyelocytic leukemia, papillary thyroid cancer, myelodysplastic syndrome, and cervical cancer (Chambon et al., 2011; Tisserand et al., 2011). In addition, TRIM25 and TRIM68 can promote the progression of breast and prostate cancer by regulating the activation of nuclear and hormone receptors (Figure 2) (Miyajima et al., 2008; Tecalco-Cruz et al., 2021).

## 5 The role of TRIMs in tumorigenesis

### 5.1 TRIMs and tumor proliferation

Excessive proliferation of tumor cells is an important feature during the process of tumor occurrence and development, and is the key target of tumor treatment (Intlekofer and Finley, 2019). TRIMs have been found to be involved in tumor proliferation. For example Jianxiong Ji et al. showed that knockout of TRIM22 using CRISPR-Cas9 decreased the proliferation of glioblastoma cells *in vitro* and *in situ* xenotransplantation models of glioblastoma, while overexpression of TRIM22 exerted the opposite effects (Ji et al., 2021). TRIM11 is highly expressed in ovarian cancer tissues, and knockout of TRIM11 induces apoptosis of ovarian cancer cells, which indicates that TRIM11 promotes the proliferation of ovarian cancer cells (Chen Y. et al., 2017). In addition, β-catenin signal-activated TRIM11 can promote the proliferation, migration, and invasion of gastric cancer (Lan et al., 2021). Another group showed that TRIM26 expression was increased in human bladder cancer tissues and cell lines, and knocking down TRIM26 significantly inhibited the proliferation, migration, and invasion of bladder cancer cells through the Akt/GSK3β/β-catenin pathway (Xie et al., 2021). Furthermore, some other TRIMs have been reported to be engaged in tumor cell proliferation; for example, SIX3 is ubiquitinated and degraded by TRIM27, and TRIM27-SIX3 signaling induces cell proliferation and metastasis in NSCLC (Liu et al., 2020). TRIM14 can accelerate the progression of the cell cycle by activating the Akt signaling pathway (Xu et al., 2017; Diao et al., 2020); TRIM47 levels were significantly upregulated in breast cancer tissues and cells, and TRIM47 knockdown significantly inhibited cell proliferation, migration, and invasion by suppressing the PI3K/Akt pathway (Wang Y. et al., 2020). Besides, TRIM44 promotes the transition from G1 phase to S phase by activating the mTOR signaling pathway and thus enhances the proliferation of various malignant tumors (Xiong et al., 2018; Li et al., 2019; Wei et al., 2019). TRIM29 can bind to TIP60 and thus inhibit the acetylation of p53k120 mediated by TIP60, subsequently enhancing cell proliferation and malignant transformation (Sho et al., 2011).

These above studies have shown that TRIMs can promote tumor proliferation; however, a small number of studies have revealed that some TRIMs act as tumor suppressors, such as Rong W and others have shown that TRIM35 inhibits the proliferation of breast cancer by enhancing the ubiquitination of PDK1 and inactivating AKT signaling pathway (Wang R. et al., 2022). Taken together, these results suggest that TRIMs are tightly involved in tumor proliferation (Table 2) (Supplementary Table S2).

**TABLE 2:** Relationship between TRIMs and tumor proliferation.

Name	Type of cancer	Mechanism	Effect on tumor proliferation	References
TRIM22	Glioblastoma	Ubiquitination accelerates the degradation of IκBα, activates NF-κB signal transduction and promotes the proliferation of glioblastoma	Promote	Ji et al. (2021)
TRIM11	Ovarian cancer	Knockdown of TRIM11 affects the expression of cell apoptosis-related (Bcl-2 and Bax) and invasion-related proteins (MMP-2 and MMP-9), reduced the phosphorylation levels of ERK and AKT	Promote	Chen et al. (2017)
	Stomach cancer	TRIM11 overexpression increases β-catenin protein levels and its downstream proteins such as CyclinD1 and C-myc	Promote	Lan et al. (2021)
TRIM26	Bladder cancer	Knockdown of TRIM26 significantly decreased the levels of p-Akt, p-GSK3β, β-catenin, and c-Myc	Promote	Xie et al. (2021)
TRIM27	Non-small cell lung cancer	SIX3 was ubiquitinated and degraded, and proliferation and metastasis were induced by SIX3-β-catenin signal transduction	Promote	Liu et al. (2020)
TRIM14	Cervical cancer; Osteosarcoma	TRIM14 induces epithelial mesenchymal transformation, cyclin D1 is upregulated and AKT signaling pathway is activated	Promote	Diao et al. (2020), Xu et al., 2017
TRIM47	Breast cancer	TRIM47 knockdown markedly inhibited the activation of PI3K/Akt signaling pathway	Promote	Wang et al. (2020)
TRIM44	Esophageal cancer; Melanoma; Colorectal cancer	TRIM44 is involved in the AKT/mTOR signaling pathway and its downstream targets, such as STAT3 phosphorylation or stabilization of TLR4 activation of AKT/mTOR	Promote	Xiong et al. (2018), Li et al. (2019), Wei et al. (2019)
TRIM29	Ataxia- telangiectasia	TRIM29 binds to Tip60, thereby inhibiting tip60-mediated acetylation of P53K120	Promote	Sho et al. (2011)
TRIM35	Breast cancer	AKT signaling is inactivated by increased ubiquitination of PDK1	Inhibit	Wang et al. (2022)

### 5.2 TRIMs and tumor metastasis

Metastasis is the main cause of tumor-related death (Lin et al., 2019). The metastatic process involves several key steps and the underlying mechanism is complex, among which the process of epithelial-mesenchymal transition (EMT) plays a critical role as EMT process engages in the decomposition of tumor extracellular matrix, destruction of cell polarity, the initiation of interstitial transcription program, and so on (Yeung and Yang, 2017; Singh et al., 2018; Bakir et al., 2020). Plenty of evidences have revealed that TRIMs can regulate tumor metastasis by promoting EMT process and the related signaling pathways.

Zhou Z and others found that TRIM44 is highly expressed in human papillary thyroid carcinoma and increases the proliferation and invasion of papillary thyroid cancer cells by activating the Wnt/β-catenin signaling pathway (Zhou et al., 2017). TRIM44 was also found to facilitate the EMT process by activating the Akt/mTOR pathway and thus promoting the metastasis of human esophageal cancer cells (Xiong et al., 2018). Another study indicated that TRIM37 can promote the migration and invasion of glioma cells by activating the PI3K/Akt signaling pathway (Tang et al., 2018) and enhance colon cancer metastasis by inducing EMT process (Hu and Gan, 2017). Similarly, knocking down TRIM47 inhibits the migration and invasion of breast cancer by suppressing PI3K/Akt pathway (Wang et al., 2020). In addition, TRIM59 can suppress the invasion, migration, and EMT process in bladder cancer by inhibiting the TGF-β/Smad2/3 signaling pathway (Chen et al., 2017); TRIM67 promotes the proliferation, migration, and invasion of NSCLC by positively regulating the Notch pathway (Jiang J. et al., 2020); and TRIM66 knockout inhibits the proliferation, migration, and invasion of colorectal cancer cells by inactivating the JAK2/STAT3 pathway (He et al., 2019). Other TRIMs, such as TRIM15 (Zhang et al., 2021), TRIM11 (Zhang et al., 2017; Lan et al., 2021) and TRIM24 (Jiang et al., 2020; Zhang et al., 2022), have also been proved to be closely related to the EMT process, leading to tumor metastasis. Notably, TRIMs can regulate tumor metastasis by directly targeting EMT executors, such as SNAIL (Table 3) (Supplementary Table S3) (Ma et al., 2018; Li et al., 2021).

**TABLE 3:** Relationship between TRIMs and tumor metastasis.

Name	Type of cancer	Mechanism	Effect on tumor metastatic	References
TRIM44	Human papillary thyroid carcinoma (PTC)	Silencing of TRIM44 inhibits the proliferation, migration and invasion of PTC cells in part through suppression of the Wnt/β-catenin signaling pathway	Promote	Zhou et al. (2017)
	Esophageal cancer	EMT is promoted through the AKT/mTOR signaling pathway and its downstream targets, such as STAT3 phosphorylation	Promote	Xiong et al. (2018)
TRIM37	Glioma cells	Knockdown of TRIM37 significantly reduced the levels of phosphorylated PI3K and Akt in U87MG cells, thus inhibiting the metastasis of glioma cells	Promote	Tang et al. (2018)
	Colon cancer	Invasion and metastasis of CRC are enhanced by the epithelial-mesenchymal transformation pathway.	Promote	Chen et al. (2017)
TRIM59	Bladder cancer	Silencing TRIM59 inhibits invasion/migration and epithelial-to-mesenchymal transition via TGF-β/Smad2/3 signaling pathway in bladder cancer cells	Promote	Wang et al. (2020)
TRIM47	Breast cancer	TRIM47 knockdown markedly inhibited the activation of PI3K/Akt signaling pathway	Promote	Jiang et al. (2020)
	Non-small cell lung cancer	Positively regulating the Notch pathway	Promote	He et al. (2019)
TRIM66	Colorectal cancer	Knockdown of TRIM66 inhibits cell proliferation, migration, and invasion in colorectal cancer through JAK2/STAT3 pathway	Promote	
TRIM15	Esophageal squamous cell carcinoma	Knockout of TRIM15 inactivated the Wnt/β-catenin signaling pathway	Promote	Zhang et al. (2021)
TRIM11	gastric cancer	Activate the beta-catenin signal	Promote	Lan et al. (2021)
	Hepatocellular carcinoma (HCC)	Knockdown of TRIM11 decreased the protein expression levels of p-PI3K and p-Akt in HCC cells and thus inhibited activation of the PI3K/Akt signaling pathway		Zhang et al. (2017)
TRIM24	Epithelial ovarian cancer	Promotes phosphorylation of Akt and EMT progression	Promote	Zhang et al. (2022)
	Renal cell carcinoma	Increasing in the expression levels of MMP-2, MMP-9, fibronectin, snail, vimentin, N-cadherin, and β-catenin, inducing the EMT process		Jiang et al. (2020)

### 5.3 TRIMs and chemoresistance

Chemoresistance is considered to be a major cause of cancer treatment failure, leading to cancer recurrence and metastasis; therefore, a comprehensive understanding of the molecular pathways underlying chemoresistance is needed to design novel therapeutic approaches (Sadri et al., 2021). TRIM proteins not only regulate the p53 pathway but also participate in other p53-independent chemoresistance acquisition mechanisms, such as the PI3K/Akt/NF-κB and Wnt/β-catenin pathways. Therefore, TRIM proteins deserve investigation to better understand chemoresistance and as a strategy to improve the efficiency of anticancer therapies (Valletti et al., 2019). TRIM7 is upregulated in osteosarcoma tissues, promotes osteosarcoma cell migration and invasion by mediating the ubiquitination of breast cancer metastasis inhibitor 1 (BRMS1), and contributes to chemotherapy resistance (Zhou et al., 2020). Cisplatin resistance is the main cause of treatment failure in patients with NSCLC, which can be mediated by autophagy. A previous study indicated that knockdown of TRIM65 can attenuate autophagy and cisplatin resistance in A549/DDP cells by regulating the miR-138-5p/ATG7 axis (Pan et al., 2019). TRIM11 positively regulates the Daple/β-catenin/ABCC9 signaling pathway through P62-selective autophagic degradation of Daple to induce drug resistance in NPC. Therefore, TRIM11 is regarded as a potential diagnostic marker and therapeutic target of chemotherapy-resistant NPC (Zhang et al., 2020). Additionally, TRIM58 decreased DDX3 expression by mediating the ubiquitination of DDX3, the downstream effector of the P53/P21 pathway, thereby inducing doxorubicin resistance in breast cancer (Wang et al., 2022). In lung cancer, TRIM46 activates AKT/HK2 signaling by modifying PHLPP2 ubiquitination to promote glycolysis and chemoresistance, which reflects the importance of TRIM46/PHLPP2/AKT signaling in lung cancer and provides a new strategy for lung cancer treatment (Tantai et al., 2022) (Table 4).

**TABLE 4:** Relationship between TRIMs and chemoresistance.

Name	Type of cancer	Mechanism	Effect on tumor chemoresistance	Reference
TRIM7	Osteosarcoma	Promote the ubiquitination of breast cancer metastasis inhibitor 1 (BRMS1)	Promote	ADDIN NE.Ref.{43C88769-6577-4C85-8A7A-6B9D4BCE28A6}(Zhou et al., 2020)
TRIM65	NSCLC	Promoting cisplatin resistance in A549/DDP cells by regulating Mir-138-5p /ATG7.	Promote	ADDIN NE.Ref.{08E35499-6045-412E-A4B2-1DC1A939C640}(Pan et al., 2019)
TRIM11	NPC	Activation of the β-catenin/ABCC9 axis by P62 selective autophagic degradation of Daple.	Promote	ADDIN NE.Ref.{E8D3D269-3174-402B-A24E-1F9695D79B21}(Zhang et al., 2020)
TRIM58	Breast cancer	Inactivation of p53/p21 by DDX3 ubiquitination in breast cancer.	Promote	ADDIN NE.Ref.{7069FE60-D253-402B-BF16-249CB7747503}(Wang et al., 2022)
TRIM46	Lung cancer	AKT/HK2 signaling is activated by modifying PHLPP2 ubiquitination.	Promote	ADDIN NE.Ref.{8D9460F1-5A44-43D8-878E-45D31E1EFEE8}(Tantai et al., 2022)

## 6 TRIMs and signaling pathways in tumors

### 6.1 TRIMs and the p53 signaling pathway

As a transcription factor, the tumor suppressor *p53* functions by transcriptionally regulating downstream target genes involved in cell cycle arrest, apoptosis, DNA repair, and metabolism. The expression level and activation of *p53* are regulated by various E3 ubiquitin ligases (such as MDM2, Pirh2, COPL, and the TRIM proteins TRIM22, TRIM24, TRIM29, and TRIM31) (Liu et al., 2021).

TRIM8 was expressed at low levels in renal clear cell carcinoma with drug resistance and wild-type p53 expression, and re-expressing TRIM8 restored chemotherapeutic sensitivity to the p53 signaling pathway and reactivated it (Caratozzolo et al., 2014), suggesting that inhibiting TRIM8 expression may inhibit p53 activity in renal clear cell carcinoma. Similarly, *TRIM22* was identified as a direct target gene of p53, and its expression level in Wilms tumors was negatively correlated with recurrence rate (Zirn et al., 2006). In another study, p53 was shown to regulate the expression of some defective *TRIM22* genes in breast cancer, and the lack of p53-mediated induction contributed to the downregulation of *TRIM22* (Sun et al., 2013; Li L. et al., 2018). Additionally, TRIM31, which is downregulated in breast cancer, inhibits the development of breast cancer by directly interacting with p53 and induces the K63 ubiquitination of p53 through its ring domain. Meanwhile, TRIM31 inhibits MDM2-mediated K48 ubiquitination by competitively inhibiting the MDM2-p53 interaction, resulting in the stability and activation of p53, thus inhibiting the proliferation, migration, and invasion of breast cancer cells (Guo et al., 2021).

In contrast, some TRIMs facilitate tumor progression by suppressing p53 activity; for example, acetylated TRIM29 can interact with p53 to change the nuclear localization of p53, thereby inhibiting p53 activity and promoting cell proliferation (Yuan et al., 2010). In addition, yeast two-hybrid experiments showed that TRIM29 interacts with the acetyltransferase Tip60 to promote Tip60 degradation and reduce the Tip60-mediated acetylation level at the P53K120 site, subsequently promoting cell proliferation and malignant transformation (Sho et al., 2011).

**FIGURE 3:**
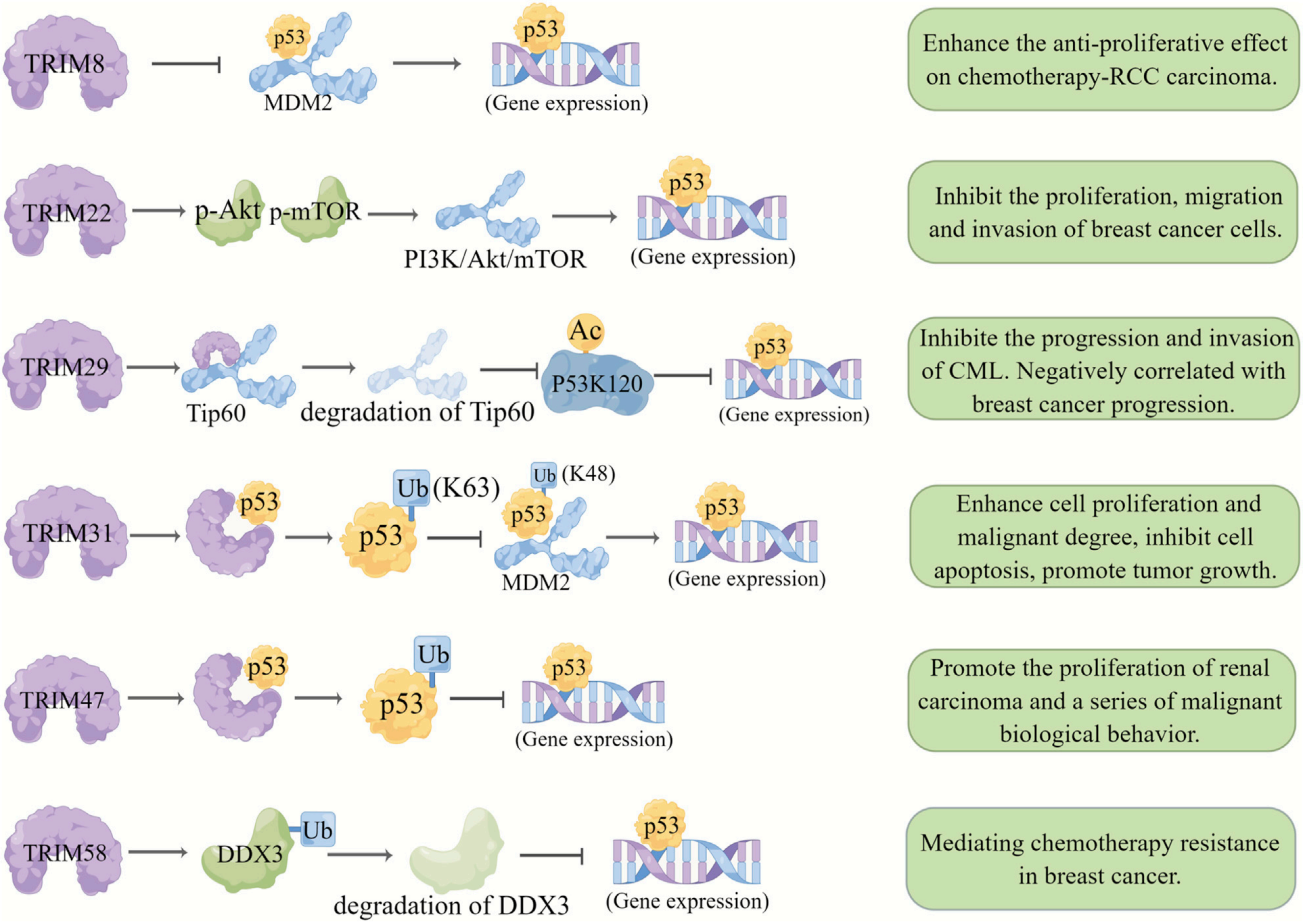
Relationship between TRIMs and p53. TRIM proteins regulate the p53 pathway by affecting different target genes and play different roles in different cancers. Degradation of a target gene is represented by a lighter color of the same pattern.TRIM8 inhibited the binding of p53 and MDM2 and stabilized the expression of p53. TRIM22 promotes the activation of the PI3K/AKT/mTOR pathway and the expression of p53 by promoting the levels of p-Akt and p-mTOR. TRIM29 interacts with the acetyltransferase TIP60 to promote the degradation of TIP60 and reduce TIP60-mediated acetylation of p53K120. TRIM31 directly interacts with p53 to induce K63 ubiquitination of p53 and inhibits MDM2-mediated K48 ubiquitination through competitive inhibition of the interaction between MDM2 and p53, leading to the stabilization and activation of p53. The interaction between TRIM47 and the p53 protein increases the ubiquitination of p53resulting in its degradation. TRIM58 interacts with DDX3, resulting in ubiquitination and degradation of DDX3 and inhibition of p53 expression.

Another TRIMs family protein TRIM47 interacts with P53 protein to increase P53 ubiquitination and degradation, thus promoting the progression of renal cell carcinoma *in vivo* and *in vitro* (Chen et al., 2021). Besides, it was found that TRIM58 interacts with DDX3 and negatively regulates the expression of DDX3, leading to ubiquitination degradation of DDX3 and thus increasing adriamycin resistance in breast cancer cells (Wang et al., 2022). These results suggest that these p53-suppressing TRIMs can be potential targets for breast cancer, especially for chemoresistant types (Figure 3) (Supplementary Table S4).

### 6.2 TRIMs and the NF-κB signaling pathway

Nuclear transcription factor-κB (NF-κB) is one of the most important nuclear transcription factors mediating intracellular signal transmission and is continuously activated in leukemias and solid tumors, including breast cancer, rectal cancer, pancreatic cancer, prostate cancer, and melanoma (Karin and Greten, 2005; Karin, 2006). NF-κB-mediated signal transduction can be activated by various cell surface or intracellular receptors, and the activation of NF-κB is strictly regulated by several PTMs including ubiquitination (Dolcet et al., 2005), through which TRIM family protein members can also regulate the NF-κB signaling pathway. Additionally, the proinflammatory cytokine tumor necrosis factor (TNF) can regulate NF-κB signaling under many physiological and pathological conditions. The expression of TRIM9, TRIM21, and TRIM62 is upregulated in the presence of TNF, which can also activate the NF-κB signaling pathway, thus these TRIMs can be used as a positive regulator of NF-κB signaling pathway (Yang and Xia, 2021). For example, TRIM22 binds to NF-κB inhibitor *α* (IκBα) and accelerates IκBα degradation by inducing K48-linked ubiquitination, leading to NF-κB activation. It also activates the NF-κB signaling pathway by activating the IKK complex and phosphorylating IκBα to release NF-κB (Qiu et al., 2015; Ji et al., 2021). TRIM27 induces IκBα ubiquitination and inhibits IκBα activity by interacting with IκBα, leading to NF-κB activation and thereby promoting the growth of human renal cell carcinoma (Xiao et al., 2021). In addition, TRIM46 can inducing the ubiquitination of PPAR to activate the NF-κB signaling pathway, increasing cell viability and inhibiting the apoptosis of osteosarcoma cells, so TRIM46 is considered a potential oncomarker of osteosarcoma (Jiang et al., 2020). TRIM44 knockdown can substantially attenuate the TNFα-dependent phosphorylation of the p65 subunit of NF-κB and IκBα, thus promoting cell proliferation and migration in breast cancer (Kawabata et al., 2017). Some other members of the TRIM family also activate NF-κB signaling through their unique domain-specific pathways; for example, TRIM52 is a novel nonclassical antiviral protein with a unique amplifying ring domain that can specifically activate NF-κB signaling dependent on its ring domain (Fan et al., 2017).

**FIGURE 4:**
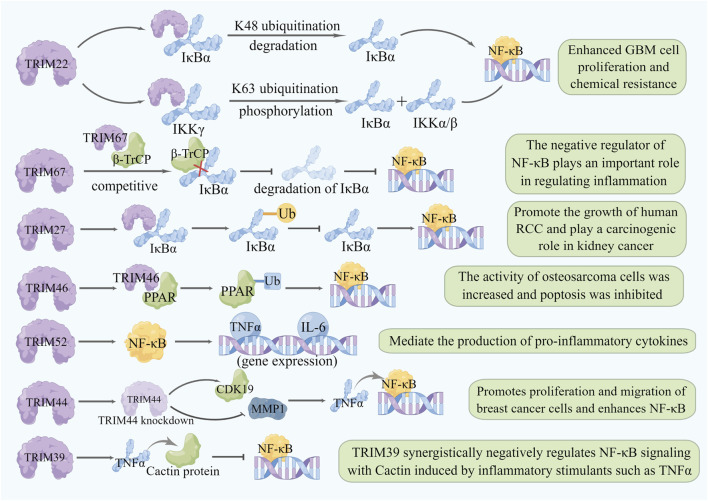
Relationship between TRIMs and NF-κB. TRIM proteins regulate the NF-κB pathway by affecting different target genes and play different roles in different cancers. Degradation of a target gene or protein knockdown is represented by a lightening of the color of the same pattern.TRIM22 binds to IκBα to accelerate the degradation of K48-linked ubiquitination, and forms a complex with IKKγ to promote the ubiquitination of K63 linked ubiquitination, which leads to the phosphorylation of IKKα/β and IκBα and activates the expression of NF-κB. TRIM67 binds to β-TrCP, competitively inhibits the binding of β-TrCP and IκBα, inhibits the degradation of IκBα, and inhibits the expression of NF-κB. TRIM27 interacts with IκBα to ubiquitinate it and inhibit its activity, leading to the activation of NF-κB. TRIM46 can interact with PPAR and activate the NF-κB signaling pathway through ubiquitination of PPAR. TRIM52 specifically activates NF-κB signaling and promotes the expression of TNFα and IL-6. TRIM44 knockdown upregulated CDK19, downregulated MMP1, and impaired TNFα-stimulated NF-κB-mediated transcriptional activity. TRIM39 stabilizes cactin through TNFα and inhibits the NF-κB-mediated signaling pathway.

Notably, some TRIMs can negatively regulate NF-κB by stabilizing negative regulators of NF-κB or inhibiting TNFα expression. For example, a yeast two-hybrid screening using TRIM39 as bait identified cactin as a negative regulator of NF-κB signal transduction and also found that TRIM39 negatively regulates NF-κB signal transduction by stabilizing cactin (Suzuki et al., 2016). Ectopic expression of TRIM67 can significantly inhibit TNFα expression and TNFα-induced NF-κB activation, so TRIM67 may be involved in inflammatory regulation as a negative regulator of the NF-κB signaling pathway (Fan et al., 2022) (Figure 4) (Supplementary Table S5).

### 6.3 TRIMs and the autophagic degradation pathway

Autophagy is an important process for cells to degrade endogenous substrates through lysosomes and is highly evolutionarily conserved. Autophagy can regulate tumor proliferation, metastasis, energy metabolism, and many other aspects (Li et al., 2020). TRIMs have been reported to regulate tumor cell autophagy. In the pathogenesis of chronic obstructive pulmonary disease (COPD), TRIM16 and galectin-3 play a synergistic role in initiating lysosomal membrane permeabilization (LMP) and inducing lysosomal autophagy (a selective lysosomal autophagy process), mediating lysosomal damage (Araya et al., 2021). TRIM21 mediates the monoubiquitination and subcellular translocation of active IKKβ to autophagosomes and inhibits IKKβ-mediated NF-κB signal transduction (Niida et al., 2010; Dickson et al., 2018). TRIM39 is a positive regulator of autophagosome lysosomal fusion through its interaction with Rab7; TRIM39 promotes Rab7 activity by inhibiting its ubiquitination at the lysine 191 residu. Furthermore, TRIM39 knockdown inhibits the tumor development and autophagic flux of colorectal cancer by inhibiting the activity of Rab7 and the autophagic degradation of p53 (Hu et al., 2021). In addition, TRIM63 interacts with P62 to regulate the conversion of nicotine to the acetylcholine receptor, increase turnover of muscular CHRN in a TRIM63-dependent manner, and produce CHRN endonuclease/lysosomal vectors (Khan et al., 2014). Similarly, TRIM13, which is located in the endoplasmic reticulum, can induce autophagy during endoplasmic reticulum stress through interaction of its coil region with p62 (Tomar et al., 2012). Furthermore, some TRIM proteins (such as TRIM23 and TRIM41) are necessary for viruses to induce autophagy, indicating that these TRIMs may be the core components of autophagy or part of the autophagy–induced pathway (Table 5) (Devkota et al., 2016; Sparrer et al., 2017).

**TABLE 5:** Relationship between TRIMs and autophagy.

Name	Target	Mechanism	Effect	References
TRIM16	Lysosomal membrane permeation	TRIM 16 and Galectin-3 synergistically recognize LMP, induce lysosomal selective autophagy, and mediate lysosomal damage	Lysosome dysfunction and hemolysis impairment associated with accelerated cell senescence	Araya et al. (2021)
TRIM21	Fc receptor	TRIM21 signaling is inhibited by its B-box domain and activated by phosphorylation. B-box occupies E2 binding sites in the catalytic ring domain by simulating E2-E3 interactions, inhibiting TRIM21 ubiquitination and preventing immune activation.	The intracellular antibody signal is regulated, and B-box is a key regulator of RING E3 ligase activity	Niida et al. (2010), Dickson et al. (2018)
TRIM39	Rab7	TRIM39 interacts with Rab7 and promotes Rab7 activity by inhibiting its ubiquitination at lysine 191 residues. Knockdown TRIM39 also inhibits autophagy degradation of p53	Inhibition of colorectal cancer progression and autophagy flux	Hu et al. (2021)
TRIM63	p62	Interacts with P62 to regulate the conversion of nicotine to acetylcholine receptors	Increased the turnover of muscle-type CHRN in a TRIM63-dependent manner, enhanced production of endo/lysosomal carriers of CHRN	Khan et al. (2014)
TRIM13	p62/SQSTM1	TRIM13 was stable during ER stress, interacting with P62 /SQSTM1 and co-locating with DFCP1.	Regulate the initiation of autophagy and decrease the clonogenesis ability of cells during ER stress	Tomar et al. (2012)
TRIM23	TBK1	Ubiquitination of TRIM23 via K27 linkage depends on GTPase to activate TBK1	Virus-induced autophagy is mediated by activation of TBK1	Sparrer et al. (2017)
TRIM41	EI24	EI24 targets RING E3 ligase, is involved in transcription, proteolysis, cell bioenergetics, and apoptosis, and is regulated by TP53 and MTOR signaling	EI24 participates in UPS autophagy crosstalk through RING E3 ligase degradation	

### 6.4 TRIMs and other signaling pathways

Akt/protein kinase B (PKB) is a serine/threonine kinase that is involved in regulating various cellular biological responses, including proliferation, apoptosis, and glycogen metabolism. Akt is activated by phosphorylation of two key residues, threonine 308 (Thr308) and serine 473 (Ser473) (Cicenas, 2008). Abnormal AKT overexpression or activation has been observed in many cancers, including ovarian, lung and pancreatic cancers, and is associated with increased cancer cell proliferation and survival. Therefore, targeting AKT may be an important approach for cancer prevention and treatment (Song et al., 2019). Some current studies suggest that the TRIM family may directly or indirectly regulate AKT signaling. Xiaojuan Xie et al. showed that TRIM26 knockdown significantly reduced the levels of p-Akt, phosphorylated glycogen synthase kinase 3β (GSK-3β) (p-GSK3β), β-catenin, and c-Myc in bladder cancer cells, and inhibited the proliferation, invasion and migration of bladder cancer cells through the Akt/GSK3β/β-catenin pathway (Xie et al., 2021). TRIM44 significantly enhances the proliferation, migration, and invasion of human esophageal cancer cells by participating in the AKT/mTOR signaling pathway and phosphorylation of its downstream target STAT3 (Xiong et al., 2018). TRIM47 knockdown inhibited the occurrence and development of breast cancer by suppressing the PI3K/Akt pathway, which was reversed by treatment with the PI3K/Akt activator insulin-like growth factor-1 (IGF-1) (Wang et al., 2020). TRIM32 is a proliferation and anti-apoptotic factor that participates in the AKT pathway of gastric cancer cells and promotes cell growth by enhancing the activity of AKT and glucose transport (Wang et al., 2020). TRIM27 promotes EMT and activates phosphorylated AKT serine/threonine kinases in colorectal cancer cells, promoting cell proliferation, invasion and migration, and inhibiting cell apoptosis (Zhang et al., 2018). Knockdown of TRIM31 reduces the expression of MMP2, MMP9, and p-Akt through the PI3K/Akt signaling pathway, thus suppressing the proliferation and invasion of gallbladder cancer cells (Li et al., 2018). Knockdown of TRIM59 inhibited the proliferation and colony formation of bile duct cancer cells *in vitro* and *in vivo* by suppressing the PI3K/AKT/mTOR signaling pathway (Shen et al., 2019). In addition, other studies have shown that TRIM59 promotes cell proliferation, invasion, and migration in colorectal cancer through the PI3K/AKT pathway (Sun et al., 2017).

Additionally, the mitogen-activated protein kinase (MAPK) pathway is another important pathway of cell signal conversion. Members of the MAPK pathway act as downstream effectors of many growth factor receptors, and this pathway is one of the most important pathways contributing to cell proliferation. There are three main subfamilies: extracellular signal-regulated kinase (ERK MAPK), C-Jun N-terminal kinase or stress-activated protein kinase (JNK or SAPK), and MAPK14 (Fang and Richardson, 2005). The MAPK signaling pathway regulates numerous biological processes, including cell proliferation, apoptosis, differentiation, the stress response and the inflammatory response, and its activation plays an important role in the occurrence and development of cancer (Braicu et al., 2019). Various studies have shown that members of the TRIM family regulate tumor progression through the MAPK pathway. The expression of TRIM11 was found to be significantly increased in ovarian cancer tissues, and knocking down TRIM11 affected the expressions of apoptosis-related proteins (Bcl-2 and Bax) and invasion-related proteins (MMP-2 and MMP-9) and reduced the phosphorylation levels of ERK and AKT (Chen et al., 2017). The activity of ERK and PI3K/AKT was also significantly inhibited by TRIM11 knockdown in lung cancer cells, which promoted cell growth, migration, and invasion (Wang et al., 2016). In human melanoma cells, TRIM45 directly interacts with activated C kinase (RACK1) to negatively regulate MAPK signaling, decreasing the activation of the MAPK signaling pathway (Sato et al., 2015). Overexpression of TRIM44 can activate MAPK signal transduction, which can induce EMT and apoptosis resistance to promote the development of intrahepatic cholangiocarcinoma (Peng et al., 2018).

**FIGURE 5:**
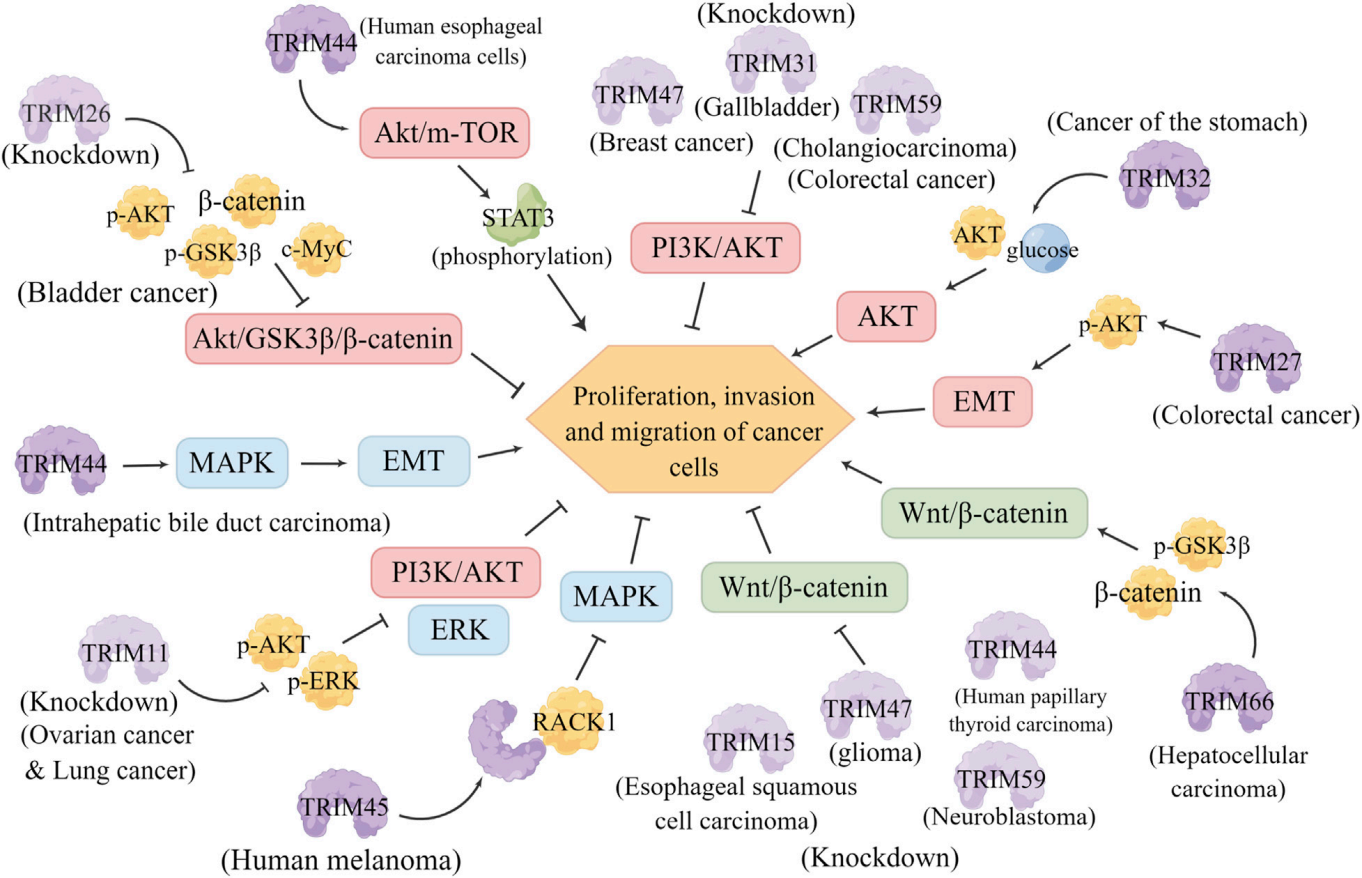
TRIMs in other signaling pathways. Relationship between TRIM family members and the AKT, MAPK, Wnt/β-catenin signaling pathways in different tumors and their regulatory mechanisms. Knockdown of TRIM proteins is indicated by a lightened color of the same pattern. AKT signaling pathway (red): Knockdown of TRIM26 significantly reduced the levels of p-Akt, p-GSK3β, β-catenin and c-Myc in bladder cancer cells, and inhibited the proliferation, invasion and migration of bladder cancer cells by inhibiting the Akt/GSK3β/β-catenin pathway. TRIM44 is involved in the phosphorylation of the AKT/mTOR signaling pathway and its downstream target gene STAT3, which significantly promotes the proliferation, migration and invasion of human esophageal cancer cells. Knockdown of TRIM47, TRIM31 and TRIM59 inhibited tumorigenesis and development by inhibiting the PI3K/AKT pathway. TRIM32 promotes cell growth by enhancing AKT activity and glucose transport. TRIM27 promotes EMT and activates p-Akt to promote cell proliferation, invasion and migration.

Furthermore, Wnt induces several intracellular signal transduction pathways, especially the Wnt/β-catenin-dependent pathway, which is involved in many important cellular functions, such as stem cell regeneration and organogenesis (Krishnamurthy and Kurzrock, 2018). The Wnt/β-catenin pathway is also considered one of the major signaling pathways involved in EMT and the regulation of cell proliferation and apoptosis, thus mediating the occurrence and progression of cancer (Arend et al., 2013). Knockdown of TRIM15 in esophageal squamous cell carcinoma (ESCC) cells significantly inhibited the proliferation, migration and invasion of cells by suppressing the Wnt/β-catenin signaling pathway (Zhang et al., 2021). Knockdown of TRIM47 significantly inhibited the activation of the Wnt/β-catenin pathway in U87 and U251 cells and thus attenuated the proliferation and metastasis of glioma cells *in vitro* and *in vivo* (Chen et al., 2020). TRIM66 is involved in the regulation of GSK-3β phosphorylation and β-catenin expression and acts as an oncoprotein in HCC by activating the GSK-3β-dependent Wnt/β-catenin signaling (Fan et al., 2019). TRIM59 was found to be upregulated and positively correlated with β-catenin in neuroblastoma tissues, and knocking down TRIM59 inhibited cell proliferation by suppressing the Wnt/β-catenin signaling pathway (Chen et al., 2019). In human papillary thyroid cancer (PTC) cells, TRIM44 knockdown inhibited the proliferation, migration and invasion of PTC cells by inactivating the Wnt/β-catenin signaling pathway; and LiCl, an activator of the Wnt/β-catenin pathway, rescued the anticancer effect of TRIM44 knockdown in PTC cells (Figure 5) (Zhou et al., 2017).

## 7 Targeting options for TRIM

The emerging critical roles of TRIMs in tumor progression make them potential targets for drug design; however, addressing the activity of this class of E3s is challenging. TRIMs, as RING ligases, belong to the largest class of E3s and lack a catalytic Cys residue; therefore, the determination of an effective targeting mode cannot depend on the elimination of enzyme activity through direct covalent-inhibition of catalytic (nucleophilic) sites. In contrast, manipulating TRIM activity requires a detailed analysis of the multidomain properties of TRIMs. Identifying potential druggable structural motifs and characterizing their functions will ultimately help to define valuable targeting options within this subclass of proteins.

Notably, the active conformation of TRIMs appears to be variable. In certain circumstances, the dimerization of the RING domain is essential for their activity, as is the case for TRIM25, TRIM32, and TRIM69 (Koliopoulos et al., 2016). The dimerization of the RING domain is affected not only by two short helices identified by the N- and C-terminal regions adjacent to the core of the RING domain but also by several other factors, such as the E2-Ub conjugate, phosphorylation, and RNA binding (Koliopoulos et al., 2016). In other cases, as some TRIMs do not have the necessary elements for the dimerization of the RING domain, TRIMs exist as monomers, as is the case for Class VI TRIMs (TRIM24, TRIM28, and TRIM33) (Fiorentini et al., 2020). Furthermore, some TRIMs undergo higher-order oligomerization prior to activation, such as TRIM19 and TRIM5α, whose RING domains are tetrameric and trimeric, respectively (Fletcher et al., 2018). These results suggest that exploring the structural features and active conformations of the TRIMs RING domain to investigate whether these differences can be used for targeting is critical.

**FIGURE 6:**
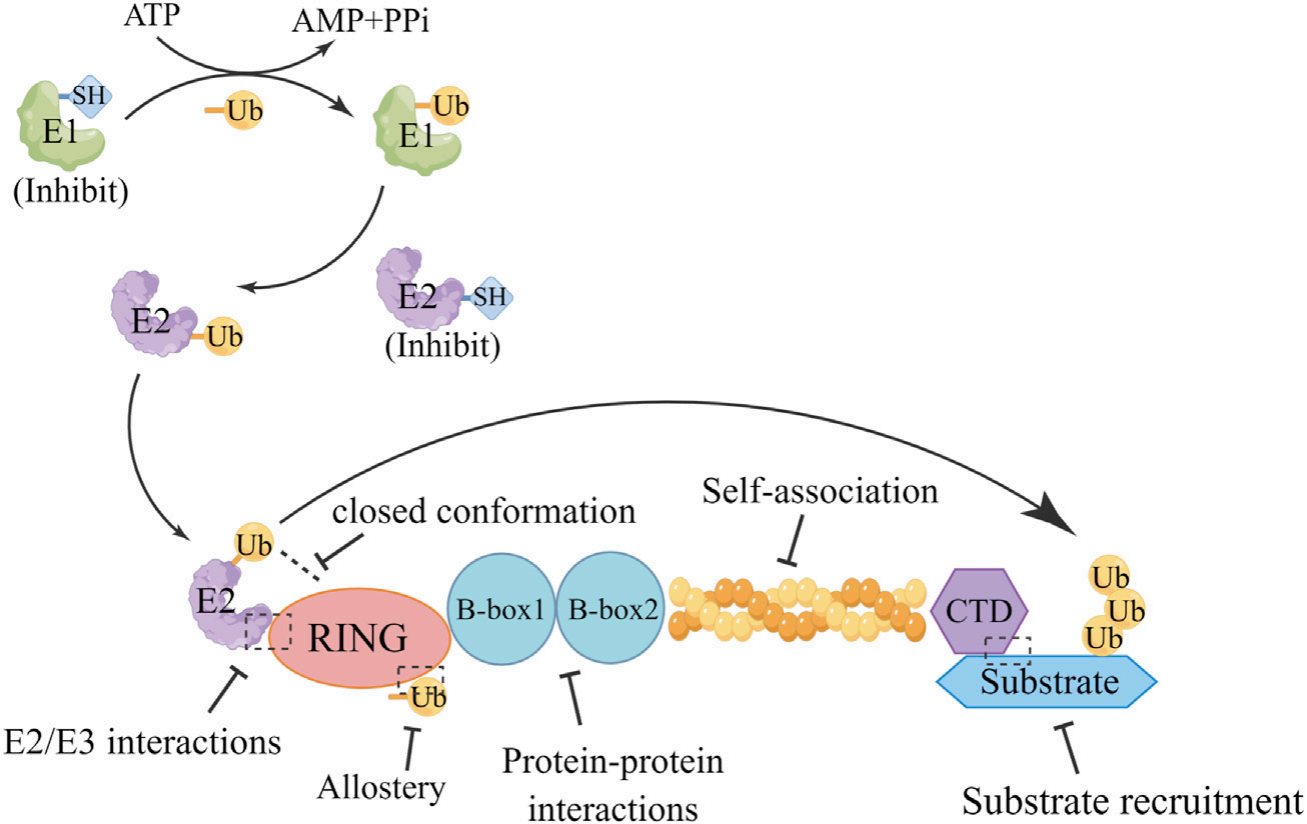
Targeting options for TRIMs. Exploring the interactions between TRIM protein domains reveals potential targeting strategies for TRIMs, which may be applied to the design of drugs targeting TRIMs.There are several strategies to consider for drugs targeting TRIMs, Targeted drugs were designed through E2/E3 interactions, closed conformations, allostery, protein-protein interactions, self-association, and substrate recruitment.

Additionally, although the biological roles of the B-Box domains in TRIMs are not yet fully understood, their function has been confirmed to be crucial in TRIM activity; for example, the B-Box 2 domain of TRIM21 autoinhibits E3 activity by occluding the E2-binding surface of the RING domain (Massiah et al., 2006). It was found that B-Box can drive higher-order oligomerization for TRIM5α and TRIM19 (Wagner et al., 2016). Intriguingly, E3 ligase activity is retained in TRIM16, which lacks the RING domain, and this effect is attributable to its RING-like folded B-box domain (Bell et al., 2012). However, it must be noted that this is not a common feature of TRIMs, as the absence of ligase activity in TRIM66 has been shown to be correlated with RING domain loss (Stoll et al., 2019). Currently, the functions of the B-Box domain have not been thoroughly studied, and further research is needed. Furthermore, the coil-coil domain has been demonstrated to be a platform for macromolecular interactions, facilitating the recruitment of cellular partners regulated by specific environmental conditions (Fonti et al., 2019). Thus, thoroughly exploring this interaction might shed light on a potential targeting strategy for TRIMs, and provide the opportunity to selectively target the oligomerization state occurring among different TRIMs by functionally characterizing this domain. Notably, targeted protein degradation (TPD) is an emerging strategy to drive the activity of RING E3 ligases toward therapeutic application and is also used as a chemical biology tool for target validation (Nabet et al., 2018). TPD can be utilized for TRIM-targeted drug design. Furthermore, the multidomain nature/multifunctional biological role of TRIMs implies that blocking just one function or one domain might not be sufficient to obtain the desired therapeutic outcome, and designing a ligand for any druggable region of a TRIM domain, independent from its function, could identify therapeutically active degraders (Figure 6). Notably, developing TRIM inhibitors for cancer therapy is currently gaining pharmaceutical attention (Zhao et al., 2021), and researchers’ growing interest in TRIM proteins has given an impetus to developing some potential drug designs shown in Table 6.

**TABLE 6:** Representative drug designs targeting TRIM proteins.

Compound name	Target	Types	Chemical formula	Activity	Cancer Type	Reference
Compound 34	TRIM24	Inhibitor	C_25_H_27_N_3_O_6_S	Kd = 222 nM	NA	ADDIN NE.Ref.{B40DD264-BD48-4609-ADBF-5AFD1DCD1DBB}(Bennett *et al.*, 2016)
IACS-6558	TRIM24	Inhibitor	C_24_H_29_N_5_O_5_S	Kd in mM level	NA	ADDIN NE.Ref.{969540FA-BFAD-4AEA-8BFA-CF3306850978}(Zhan *et al.*, 2015)
				Kd = 31I nM		
IACS-9571	TRIM24	Inhibitor	C_32_H_42_N_4_O_8_S	EC50 = 50 nM		ADDIN NE.Ref.{72FD7007-6D2A-4F44-8206-C6E2CC818A65}(Palmer *et al.*, 2016)
	NA			IC50 = 8 nM		
dTRIM24	TRIM24	Degrader	C_61_H_81_O_14_N_9_S_2_	NA	Glioblastoma	ADDIN NE.Ref.{2BAAF6F3-CEDD-40EA-B1D8-4035B3D4AAA7}(Gechijian *et al.*, 2018; Han and Sun, 2021)
Arsenic trioxide	TRIM19	Degrader	AS_2_O_3_	NA	Glioma	ADDIN NE.Ref.{54799D50-F28E-40B8-AADC-D7743CFA437E}(Zhou *et al.*, 2015)
Red orpiment	TRIM19	Inhibitor	As_2_H_6_S_3_	NA	Acute promyelocytic leukemia	ADDIN NE.Ref.{73C4D97A-E4D0-4760-9C84-099C858D406A}(Zhong *et al.*, 2003)
Genistein	TRIM19	Inhibitor	C_15_H_10_O_5_	NA	Acute promyelocytic leukemia	ADDIN NE.Ref.{3752BF82-6CD0-49F3-AF41-655F1E03BC99}(Ng *et al.*, 2007)
Dexamethasone	TRIM19	Inhibitor	C_22_H_29_FO_5_	NA	Acute B-lymphocytic leukemia	ADDIN NE.Ref.{CAE8DE67-050C-4BEC-9C4C-EFC525A0DF90}(Laane *et al.*, 2009)
2,5-MeC	TRIM19	Inhibitor	C_11_H_12_O_4_	NA	Lung cancer and osteosarcoma	ADDIN NE.Ref.{192516F9-5E36-4764-A10B-6E009323E0E1}(Komura *et al.*, 2007)
Sodium arsenite	TRIM19	Inhibitor	AsNaO_2_	NA	Hepatocellular carcinoma	ADDIN NE.Ref.{9E4D1B65-990C-4F10-9055-A07054EAE196}(Tang *et al.*, 2016)
Withaferin A	TRIM16	Inhibitor	C_28_H_38_O_6_	NA	Melanoma	ADDIN NE.Ref.{E99FEC92-EE87-4452-810A-AFA675EDE23F}(Nagy *et al.*, 2020)
Chanti-TRIM	TRIM14	Inhibitor	NA	10-160 mg/ml	osteosarcoma	ADDIN NE.Ref.{BC475E65-AA35-410B-91C0-01F564ED1C4D}(Li *et al.*, 2018)
Verteporfin	TRIM28	Inhibitor	C_82_H_84_N_8_O_16_	NA	Melanoma and lung cancer	ADDIN NE.Ref.{DCEBE494-5243-4B05-BF01-7A9C6537D7F9}(Liang *et al.*, 2020)
Eugenol	TRIM59	Inhibitor	C_10_H_12_O_2_	NA	Lung caner	ADDIN NE.Ref.{CBE5BEB1-7E16-4435-A272-E5D6B69259DF}(Cui *et al.*, 2019)
GA-13315	TRIM67	Inhibitor	C_19_H_17_C_l_O_6_	NA	Lung caner	ADDIN NE.Ref.{BA2454FC-3E44-4838-BBE0-F6AF06E09B00}(Liu *et al.*, 2019)

## 8 Conclusion

In summary, TRIMs, as a class of E3 ubiquitin ligases, play an important role in the occurrence, development, and drug resistance of tumors. They can regulate various aspects of tumor development, such as the proliferation, migration, and invasion of tumor cells (Hatakeyama, 2017). From the perspective of the mechanism of TRIMs, they regulate protein ubiquitination, protein chromosome abnormalities, transcription, autophagy, and other processes mainly by affecting signaling pathways, such as the p53 signaling pathway and NF-κB signaling pathway (Ozato et al., 2008). Notably, TRIM family members can play different roles in promoting or inhibiting tumors, and different domains of TRIMs themselves can even play different roles in tumors (Esposito et al., 2017). A growing body of evidence suggests that members of the TRIM may play different or even opposite roles in different types of tumors (Zhao et al., 2021). This means that a large number of studies are needed to verify the effects of each TRIM protein in different tumors, and conditional engineered mouse models (tissue-specific knockout mouse models and transgenic mouse models) are favorable approaches for such research.

Currently, our research on the TRIM family is fragmentary, and the role of each member in tumor development and its precise regulatory mechanism are not fully understood. However, with increasing studies on TRIMs in different diseases, the role of the TRIM family in the occurrence and development of various diseases and tumors has been fully confirmed. These experimental results fully demonstrate the importance of TRIMs in diseases (Mohammadi et al., 2022). Therefore, it is necessary to further study each member of the TRIM family to elucidate its role in various diseases, especially tumors. In this paper, most of the research articles on TRIM proteins were integrated and systematically sorted, which can help readers quickly become familiar with the research on related topics and facilitate the retrieval of existing research results to facilitate further research on the role of TRIM in tumor diseases. Further research on the role of TRIMs in tumor diseases can provide new ideas for molecular diagnosis and gene therapy of malignant tumors, facilitate the identification of potential targets for the treatment of malignant tumors, and develop new therapeutic strategies in clinical practice. It can also facilitate drug discovery efforts using the current platform of reagents and assay readout, targeting all aspects of the ubiquitin cascade, and important information can be obtained from studying other classes of RING enzymes. Therefore, TRIM proteins might be eligible targets for the development of PROTACs.

Drug repurposing is an effective strategy for drug research and development that can shorten the research and development cycle, reduce costs and reduce risks. Although there is no starting point for the design of inhibitors for TRIM proteins, one promising strategy that could be useful to overcome this limitation consists of the evaluation of off-patented drugs. Interestingly, a previous study performed a small-scale screen against a panel of marketed quinoline-based drugs in MM cells and showed that nitroxoline (NXQ) was the most effective drug at suppressing MM cell proliferation (Mao et al., 2017). Mechanistically, they found that NXQ downregulated TRIM25. However, the detailed mechanisms by which NXQ downregulates TRIM25 levels are still unclear. Importantly, TRIM proteins may function as dual regulators of the immune response and carcinogenesis, and it would be important to consider the crosstalk of TRIM protein functions among immunity, cancer, and autophagy (Hatakeyama, 2017). Overall, these multifaceted roles of TRIM proteins undoubtedly represent a good reason to further investigate TRIM targeting as an integrative approach in cancer. In-depth studies of various roles of each TRIM family protein in different diseases may provide new strategies for targeted treatment of malignant tumors, solve problems in the treatment of malignant tumors, and bring progress to medical related research.

## Supplementary material

SUPPLEMENTARY FIGURE S1 Schematic diagram of literature search and inclusion process.

## Glossary

ACID: Acid rich region
AKT: Protein kinase B
ARF: ADP ribosylation factor family domain
APOA1: Apolipoprotein A1
AMPK: AMP-activated protein kinase
APL: Acute promyelocytic leukemia
BROMO: Bromine domain
BRCA1: Breast cancer susceptibility 1
BRMS1: Breast cancer metastasis-suppressor 1
β-TrCP: β-transduction repeat protein
CRC: Colorectal Cancer
COPD: Chronic obstructive pulmonary disease
CDK: Cyclin Dependent Kinase
EGFR: Epidermal Growth Factor Receptor
E1: Ubiquitin-activating Enzyme 1
E2: Ubiquitin-activating Enzyme 2
E3: Ubiquitin Ligase 3
ERK: Extracellular Signal-Regulated Kinase
EMT: Epithelial mesenchymal transition
EI24: Etoposide-induced protein 2.4
EMCV: Cerebral myocarditis virus
ESCC: Esophageal squamous cell carcinoma
GSK-3β: Glycogen synthase kinase-3β
HER2: Human Epidermal growth factor Receptor 2
HCC: Hepatocellular Carcinoma
HSV-1: Herpes simplex virus 1
IκBα: Nuclear factor of kappa light polypeptide gene enhancer in B-cells inhibitor, alpha
IAV: Influenza A virus
JAK: Janus Kinase
JNK: Jun N-terninal kinase
MAVS: Mitochondria antiviral signaling
mTOR: Mechanistic Target of Rapamycin
MMP: Matrix Metalloproteinase
mTORC1: Mammalian target of rapamycin complex 1
MDM2: Murine Double Minute 2
NEMO: NF-κB essential modulator
NSCLC: Non-Small Cell Lung Cancer
NF-κB: Nuclear factor kappa-light-chain-enhancer of activated B cells
NPC: Nasopharyngeal carcinoma
NXQ: Nitroxoline
PTM: Post-translational modification
PI3K: Phosphatidylinositol 3-Kinase
PDK1: Pyruvate dehydrogenase kinase isoenzyme 1
PTEN: Phosphatase and tensin homolog deleted on chromosome TEN
PTC: Papillary Thyroid Cancer
PML: Promyelocytic leukemia
PML-RARα: Promyelocytic leukemia-retinoic acid receptor alpha
PML-NBs: Promyelocytic leukemia-nuclear bodies
PALB2: Partner and localizer of BRCA2
PPAR: Peroxisome proliferator-activated receptor
PKB: Protein kinase B
RLRs: RIG-I like receptors
RING: Really Interesting New Gene
RCC: Renal Cell Carcinoma
RARα: Retinoic acid receptor *α*
RBR: RING1-in-between-RING2
RACK1: Receptor of activated protein C kinase 1
STAT: Signal Transducers and Activators of Transcription
SMAD: Solvated metal atoms disperse
SAPK: Stiess-activated protein kinase
TRIM: Tripartite motif
TGF: Transforming Growth Factor
TLR4: Toll-like receptor 4
TSC1: Tuberous sclerosis complex subunit 1
TSC2: Tuberous sclerosis complex subunit 2
TM: Transmembrane region
TNF: Tumor necrosis factor
TBK1: Threonine-protein kinase 1
TPD: Target protein degra dation
