# Supplementary figures and images for: The roles and targeting options of TRIM family proteins in tumor
Source: Front Pharmacol. 2022 Sep 30;13:999380. doi: 10.3389/fphar.2022.999380 (PMC9561884; doi:10.3389/fphar.2022.999380)

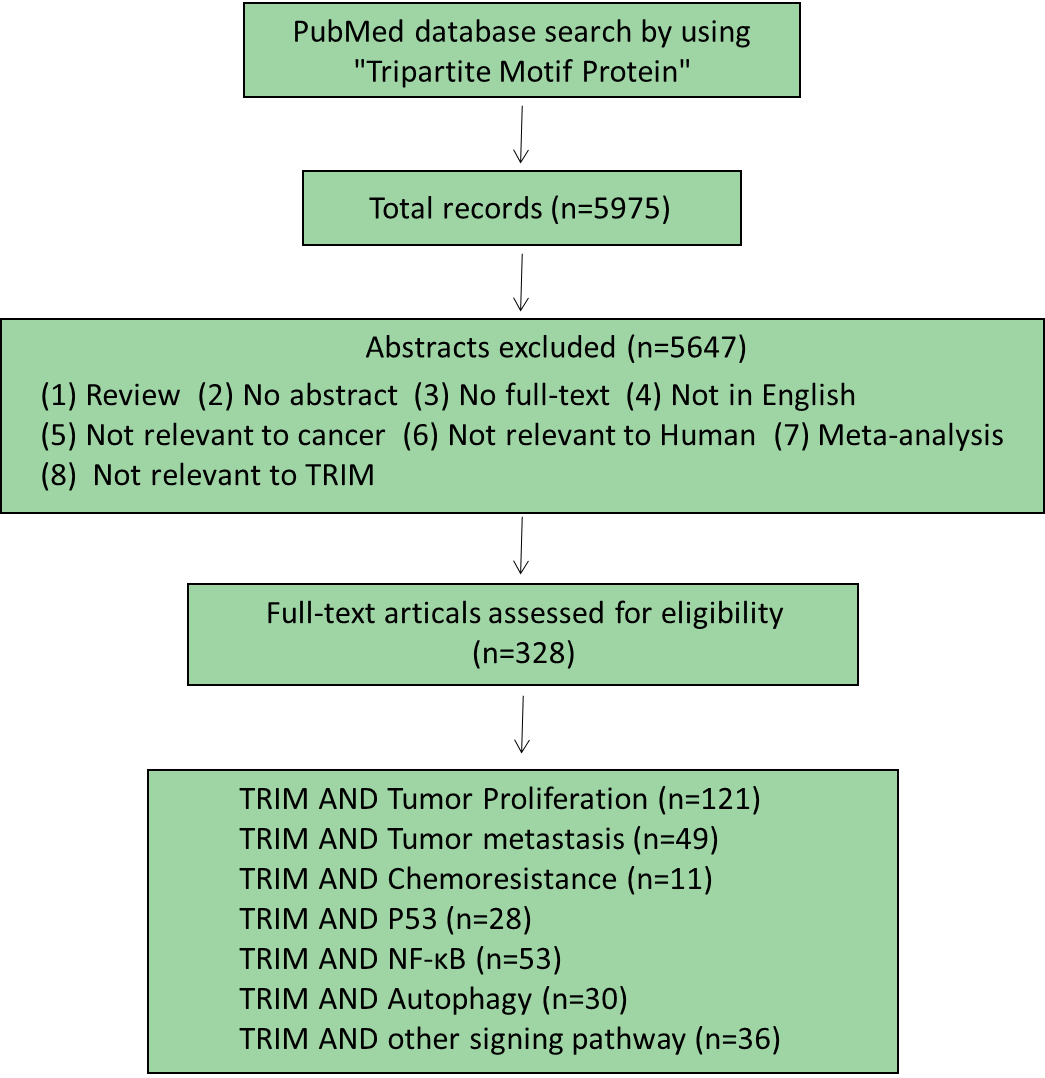

Supplement: Supplementary file 1 [file Image1.TIF]
