# Supplementary material for: The roles and targeting options of TRIM family proteins in tumor
Source: Front Pharmacol. 2022 Sep 30;13:999380. doi: 10.3389/fphar.2022.999380 (PMC9561884; doi:10.3389/fphar.2022.999380)
Supplement: Supplementary file 2 [file DataSheet1.docx]

**Supplement Table 1 Relationship between TRIMs and ubiquitination**

| Name | Ubiquitination targets | Effects | Reference |
| --- | --- | --- | --- |
| TRIM7 | Breast cancer metastasis suppressor 1 (BRMS1) | It positively regulates the tumorigenesis and chemoresistance of osteosarcoma | (Zhou *et al.*, 2020) |
| TRIM23 | TRIM23 TBK1 - p62 axis | Virus-induced autophagy is mediated by TBK1 activation | (Sparrer *et al.*, 2017) |
| TRIM21 | Ube2N/Ube2V2 | Drives broad-spectrum anti-pathogen targeting and supports protein-consuming technology TRIM-AWAY | (Kiss *et al.*, 2019) |
| TRIM35 | TRAF3 | Mediates protection against influenza infection | (Sun *et al.*, 2020) |
| TRIM22 | IκBα/IKKγ | Activation of NF-κB signaling in glioblastoma | (Ji *et al.*, 2021) |
| TRIM52 | SHP2 | Promote the proliferation of colorectal cancer cells | (Pan *et al.*, 2019) |
| TRIM31 | MAVS | It promotes the formation of prion-like aggregates of MAVS after viral infection | (Liu *et al.*, 2017) |
| TRIM25 | RIG-I | It is essential for the cellular solute RIG-I signaling pathway to trigger the host antiviral innate immunity | (Gack *et al.*, 2007) |
| TRIM41 | VSV-N | Limit vesicular stomatitis virus infection | (Patil *et al.*, 2020) |
| TRIM40 | NLRP3 | Inhibition of IGA1-induced proliferation of glomerular mesangial cells | (Shen *et al.*, 2021) |
| TRIM17  TRIM28 | BCL2A1 | Antagonism regulates BCL2A1 stability and regulates cell death | (Lionnard *et al.*, 2019) |
| TRIM47 | SMAD4 | Leading to the growth and invasion of human CRC cells | (Liang *et al.*, 2019) |
| TRIM59 | PPM1A | Activation of TGF-β/Smad signaling promotes invasion of ectopic endometrial stromal cells into endometriosis | (Wang *et al.*, 2020) |
| TRIM65 | ANXA2 | Supports the invasiveness of bladder urothelial carcinoma cells | (Wei *et al.*, 2018) |
| TRIM46 | PPAR | Promote the viability of osteosarcoma cells and inhibit cell apoptosis | (Jiang *et al.*, 2020) |
| TRIM58 | DDX3 | Mediates chemoresistance in breast cancer | (Wang *et al.*, 2022) |
| TRIM27 | NOD2 | Influence NOD2-mediated proinflammatory response | (Zurek *et al.*, 2012) |
| TRIM72 | MGMT | Increased the sensitivity of uveal melanoma cells to dacarbazine treatment | (Li *et al.*, 2022) |
| TRIM52 | PPM1A | Promote proliferation, migration and invasion | (Zhang *et al.*, 2018) |
| TRIM21 | AKT | Inhibition of osteogenic differentiation of mesenchymal stem cells | (Xian *et al.*, 2022) |
| TRIM4 | RIG-I | Modulates type I interferon induction and cellular antiviral response | (Yan *et al.*, 2014) |
| TRIM54 | FLNC | Promote gastric cancer progression | (Cao *et al.*, 2022) |
| TRIM35 | PDK1 | It can induce cell apoptosis and play a new tumor suppressor role in breast cancer | (Wang *et al.*, 2022) |
| TRIM69 | NS3 | Interruption of dengue virus replication | (Wang *et al.*, 2018) |
| TRIM6 | VP35 | Promote virus replication | (Bharaj *et al.*, 2017) |

**Supplement Table 2 Relationship between TRIMs and tumor proliferation**

| Name | Type of cancer | Mechanism | Reference |
| --- | --- | --- | --- |
| TRIM52 | Colorectal cancer | The proliferation of colorectal cancer cells was promoted by STAT3 signaling | (Pan *et al.*, 2019) |
| TRIM21 | Glioma | Regulation of cell proliferation, cell migration and cell senescence in human glioma promotes tumor progression | (Zhao *et al.*, 2020) |
| TRIM31 | Glioma | It promotes glioma proliferation and invasion by activating NF-κB pathway; AKT signaling pathway | (Zhou *et al.*, 2019; Shi *et al.*, 2019) |
|  | Gallbladder | Knockdown of TRIM31 down-regulates MMP2/9 through PI3K/Akt signaling pathway | (Li *et al.*, 2018) |
| TRIM44 | Testicular germ cell neoplasms | It can promote cell proliferation and migration and inhibit apoptosis of testicular germ cell tumor | (Yamada *et al.*, 2017) |
| TRIM24 | Renal cell carcinoma | Induction of EMT promotes tumor progression | (Jiang *et al.*, 2020) |
|  | Colorectal cancer | Regulation of colorectal cancer cell proliferation by YAP signaling | (Xie *et al.*, 2020) |
| TRIM59 | Neuroblastoma; Breast cancer; Bone sarcoma; Human hepatocellular carcinoma; Non-small cell lung cancer | TRIM59 knockdown inhibits cell proliferation by down-regulating Wnt/β-catenin signaling pathway in neuroblastoma | (Chen *et al.*, 2019; Zhang and Yang, 2017; Liang *et al.*, 2016; Sun *et al.*, 2017; Zhan *et al.*, 2015) |
| TRIM47 | Glioma | As a tumorigenesis promoter, upregulated tripartite motif 47 can promote glioma cell proliferation and metastasis | (Ji *et al.*, 2021) |
| TRIM66 | Colorectal cancer | Knockdown of TRIM66 inhibits the proliferation, migration and invasion of colorectal cancer cells through JAK2/STAT3 pathway | (He *et al.*, 2019) |
| TRIM37 | Non-small cell lung cancer | Inhibits NSCLC cell migration and invasion by inhibiting epithelial-mesenchymal transition (EMT) phenotype | (Ding *et al.*, 2018) |
| TRIM6 | Colorectal cancer | Promotes proliferation and response to thiostreptospirin in colorectal cancer cells through TIS21/FoxM1 | (Zheng *et al.*, 2020) |
| TRIM67 | Non-small cell lung cancer | Positive regulation of Notch pathway promotes the proliferation, migration and invasion of non-small cell lung cancer | (Jiang *et al.*, 2020) |
| TRIM32 | Cancer of the stomach | It promotes cell proliferation and invasion by activating β-catenin signaling in gastric cancer | (Wang *et al.*, 2018) |
| TRIM25 | Colorectal cancer | It promotes the proliferation and invasion of colorectal cancer cells through TGF-β signaling | (Sun *et al.*, 2017) |
| TRIM28 | Lung cancer | Regulation of cell proliferation by bridging HDAC1/E2F interaction | (Chen *et al.*, 2012) |
| TRIM2 | Clear cell renal cell carcinoma | It affects cell proliferation, migration and invasion | (Xiao *et al.*, 2018) |

**Supplement Table 3 Relationship between TRIMs and tumor metastasis**

| Name | Type of cancer | Mechanism | Reference |
| --- | --- | --- | --- |
| TRIM65 | Urothelial carcinoma of the bladder | Support the invasiveness of bladder urothelial cancer cells by promoting ANXA2 ubiquitination and degradation | (Wei *et al.*, 2018) |
| TRIM47 | Glioma | Knockdown of TRIM47 inhibited the proliferation, migration and invasion of glioma cells through the inactivation of Wnt/β-catenin pathway | (Chen *et al.*, 2020) |
| TRIM13 | Clear cell renal cell carcinoma | Upregulation of TRIM13 resulted in decreased levels of NF-KB, MMP-9, and P-Akt, as well as reduced migration and invasion abilities | (Li *et al.*, 2020) |
| TRIM16 | Cancer of the liver | TRIM16 knockdown promotes epithelial-mesenchymal transition (EMT) in a manner associated with HCC metastasis in vitro and in vivo | (Li *et al.*, 2016) |
| TRIM29 | Squamous cell carcinoma | Loss of TRIM29 alters keratin distribution to promote cell invasion in SCC | (Yanagi *et al.*, 2018) |
| TRIM14 | osteosarcoma | It regulates osteosarcoma cell proliferation and invasion by promoting AKT signaling pathway | (Xu *et al.*, 2017) |

**Supplement Table 4 Relationship between TRIMs and P53**

| Name | Type of cancer | Influence | Reference |
| --- | --- | --- | --- |
| TRIM67 | Colorectal cancer | TRIM67 activates p53 to inhibit the occurrence and progression of colorectal cancer | (Wang *et al.*, 2019) |
| TRIM27 | Ischemia reperfusion injury of the heart | TRIM27 prevents cardiac ischemia-reperfusion injury by negatively regulating p53 to inhibit apoptosis and inflammation | (Li *et al.*, 2021) |
| TRIM31 | Cancer of the liver | By regulating p53-AMPK axis, the resistance of hepatocellular carcinoma cells to lost-loss apoptosis was promoted | (Guo *et al.*, 2018) |
| TRIM11 | Hepatocellular carcinoma (HCC) | It plays an oncogenic role in hepatocellular carcinoma by inhibiting P53 | (Liu *et al.*, 2017) |

**Supplement Table 5 Relationship between TRIMs and NF-κB**

| Name | Type of cancer | Influence | Reference |
| --- | --- | --- | --- |
| TRIM52 | Ovarian cancer | TRIM52 plays an oncogenic role in ovarian cancer development associated with NF-KB signaling | (Yang *et al.*, 2018) |
| TRIM47 | Breast cancer | Through PKC-ε/PKD3 stable activation of NF-κB signaling, and contribute to the resistance of breast cancer to endocrine therapy | (Azuma *et al.*, 2021) |
| TRIM14 | Osteosarcoma | Nuclear factor-κB signaling pathway promotes the aggressiveness of osteosarcoma | (Li *et al.*, 2018) |
| TRIM31 | Glioma | It promotes glioma proliferation and invasion by activating NF-κB pathway | (Zhou *et al.*, 2019) |
| TRIM13 | Non-small cell lung cancer | It inhibited cell proliferation and induced cell apoptosis by regulating NF-κB pathway | (Xu *et al.*, 2019) |
| TRIM44 | Human lung cancer | It promotes the migration and invasion of human lung cancer cells through NF-κB signaling pathway | (Luo *et al.*, 2015) |

**References:**

Azuma, K., Ikeda, K., Suzuki, T., Aogi, K., Horie-Inoue, K. & Inoue, S. (2021), "TRIM47 activates NF-kappaB signaling via PKC-epsilon/PKD3 stabilization and contributes to endocrine therapy resistance in breast cancer", *Proc Natl Acad Sci U S A,* Vol. 118 No. 35, pp.

Bharaj, P., Atkins, C., Luthra, P., Giraldo, M. I., Dawes, B. E., Miorin, L., Johnson, J. R., Krogan, N. J., Basler, C. F., Freiberg, A. N. & Rajsbaum, R. (2017), "The Host E3-Ubiquitin Ligase TRIM6 Ubiquitinates the Ebola Virus VP35 Protein and Promotes Virus Replication", *J Virol,* Vol. 91 No. 18, pp.

Cao, H., Li, Y., Chen, L., Lu, Z., You, T., Wang, X. & Ji, B. (2022), "Tripartite motif-containing 54 promotes gastric cancer progression by upregulating K63-linked ubiquitination of filamin C", *Asia Pac J Clin Oncol,*.

Chen, G., Chen, W., Ye, M., Tan, W. & Jia, B. (2019), "TRIM59 knockdown inhibits cell proliferation by down-regulating the Wnt/beta-catenin signaling pathway in neuroblastoma", *Biosci Rep,* Vol. 39 No. 1, pp.

Chen, L., Chen, D. T., Kurtyka, C., Rawal, B., Fulp, W. J., Haura, E. B. & Cress, W. D. (2012), "Tripartite motif containing 28 (Trim28) can regulate cell proliferation by bridging HDAC1/E2F interactions", *J Biol Chem,* Vol. 287 No. 48, pp. 40106-18.

Chen, L., Li, M., Li, Q., Xu, M. & Zhong, W. (2020), "Knockdown of TRIM47 inhibits glioma cell proliferation, migration and invasion through the inactivation of Wnt/beta-catenin pathway", *Mol Cell Probes,* Vol. 53101623.

Ding, Y., Lu, Y., Xie, X., Sheng, B. & Wang, Z. (2018), "Silencing TRIM37 inhibits the proliferation and migration of non-small cell lung cancer cells", *RSC Adv,* Vol. 8 No. 64, pp. 36852-36857.

Gack, M. U., Shin, Y. C., Joo, C. H., Urano, T., Liang, C., Sun, L., Takeuchi, O., Akira, S., Chen, Z., Inoue, S. & Jung, J. U. (2007), "TRIM25 RING-finger E3 ubiquitin ligase is essential for RIG-I-mediated antiviral activity", *Nature,* Vol. 446 No. 7138, pp. 916-920.

Guo, P., Qiu, Y., Ma, X., Li, T., Ma, X., Zhu, L., Lin, Y. & Han, L. (2018), "Tripartite motif 31 promotes resistance to anoikis of hepatocarcinoma cells through regulation of p53-AMPK axis", *Exp Cell Res,* Vol. 368 No. 1, pp. 59-66.

He, T., Cui, J., Wu, Y., Sun, X. & Chen, N. (2019), "Knockdown of TRIM66 inhibits cell proliferation, migration and invasion in colorectal cancer through JAK2/STAT3 pathway", *Life Sci,* Vol. 235116799.

Ji, B., Liu, L., Guo, Y., Ming, F., Jiang, J., Li, F., Zhao, G., Wen, J. & Li, N. (2021), "Upregulated Tripartite Motif 47 Could Facilitate Glioma Cell Proliferation and Metastasis as a Tumorigenesis Promoter", *Comput Math Methods Med,* Vol. 20215594973.

Ji, J., Ding, K., Luo, T., Zhang, X., Chen, A., Zhang, D., Li, G., Thorsen, F., Huang, B., Li, X. & Wang, J. (2021), "TRIM22 activates NF-kappaB signaling in glioblastoma by accelerating the degradation of IkappaBalpha", *Cell Death Differ,* Vol. 28 No. 1, pp. 367-381.

Jiang, J., Ren, H., Xu, Y., Wudu, M., Wang, Q., Liu, Z., Su, H., Jiang, X., Zhang, Y., Zhang, B. & Qiu, X. (2020), "TRIM67 Promotes the Proliferation, Migration, and Invasion of Non-Small-Cell Lung Cancer by Positively Regulating the Notch Pathway", *J Cancer,* Vol. 11 No. 5, pp. 1240-1249.

Jiang, T., Mao, H., Chen, Q., Cao, L., He, Y., Gao, X., Chen, W. & Zhang, H. (2020), "Trim24 prompts tumor progression via inducing EMT in renal cell carcinoma", *Open Med (Wars),* Vol. 15 No. 1, pp. 1153-1162.

Jiang, W., Cai, X., Xu, T., Liu, K., Yang, D., Fan, L., Li, G. & Yu, X. (2020), "Tripartite Motif-Containing 46 Promotes Viability and Inhibits Apoptosis of Osteosarcoma Cells by Activating NF-B Signaling Through Ubiquitination of PPAR", *Oncol Res,* Vol. 28 No. 4, pp. 409-421.

Kiss, L., Zeng, J., Dickson, C. F., Mallery, D. L., Yang, J. C., McLaughlin, S. H., Boland, A., Neuhaus, D. & James, L. C. (2019), "A tri-ionic anchor mechanism drives Ube2N-specific recruitment and K63-chain ubiquitination in TRIM ligases", *Nat Commun,* Vol. 10 No. 1, pp. 4502.

Li, H., Qu, L., Zhou, R., Wu, Y., Zhou, S., Zhang, Y., Cheng, B., Ni, J., Huang, H. & Hou, J. (2020), "TRIM13 inhibits cell migration and invasion in clear-cell renal cell carcinoma", *Nutr Cancer,* Vol. 72 No. 7, pp. 1115-1124.

Li, H., Zhang, Y., Hai, J., Wang, J., Zhao, B., Du L & Geng, X. (2018), "Knockdown of TRIM31 suppresses proliferation and invasion of gallbladder cancer cells by down-regulating MMP2/9 through the PI3K/Akt signaling pathway", *Biomed Pharmacother,* Vol. 1031272-1278.

Li, L., Dong, L., Qu, X., Jin, S., Lv, X. & Tan, G. (2016), "Tripartite motif 16 inhibits hepatocellular carcinoma cell migration and invasion", *Int J Oncol,* Vol. 48 No. 4, pp. 1639-49.

Li, X., Yang, C., Luo, N., Yang, Y., Guo, Y., Chen, P. & Cun, B. (2022), "Ubiquitination and degradation of MGMT by TRIM72 increases the sensitivity of uveal melanoma cells to Dacarbazine treatment", *Cancer Biomark,* Vol. 34 No. 2, pp. 275-284.

Li, Y. J., Zhang, G. P., Zhao, F., Li, R. Q., Liu, S. J., Zhao, Z. R. & Wang, X. (2018), "Target therapy of TRIM-14 inhibits osteosarcoma aggressiveness through the nuclear factor-kappaB signaling pathway", *Exp Ther Med,* Vol. 15 No. 3, pp. 2365-2373.

Li, Y., Meng, Q., Wang, L. & Cui, Y. (2021), "TRIM27 protects against cardiac ischemia-reperfusion injury by suppression of apoptosis and inflammation via negatively regulating p53", *Biochem Biophys Res Commun,* Vol. 557127-134.

Liang, J., Xing, D., Li, Z., Shen, J., Zhao, H. & Li, S. (2016), "TRIM59 is upregulated and promotes cell proliferation and migration in human osteosarcoma", *Mol Med Rep,* Vol. 13 No. 6, pp. 5200-6.

Liang, Q., Tang, C., Tang, M., Zhang, Q., Gao, Y. & Ge, Z. (2019), "TRIM47 is up-regulated in colorectal cancer, promoting ubiquitination and degradation of SMAD4", *J Exp Clin Cancer Res,* Vol. 38 No. 1, pp. 159.

Lionnard, L., Duc P, Brennan, M. S., Kueh, A. J., Pal, M., Guardia, F., Mojsa, B., Damiano, M. A., Mora, S., Lassot, I., Ravichandran, R., Cochet, C., Aouacheria, A., Potts, P. R., Herold, M. J., Desagher, S. & Kucharczak, J. (2019), "TRIM17 and TRIM28 antagonistically regulate the ubiquitination and anti-apoptotic activity of BCL2A1", *Cell Death Differ,* Vol. 26 No. 5, pp. 902-917.

Liu, B., Zhang, M., Chu, H., Zhang, H., Wu, H., Song, G., Wang, P., Zhao, K., Hou, J., Wang, X., Zhang, L. & Gao, C. (2017), "The ubiquitin E3 ligase TRIM31 promotes aggregation and activation of the signaling adaptor MAVS through Lys63-linked polyubiquitination", *Nat Immunol,* Vol. 18 No. 2, pp. 214-224.

Liu, J., Rao, J., Lou, X., Zhai, J., Ni, Z. & Wang, X. (2017), "Upregulated TRIM11 Exerts its Oncogenic Effects in Hepatocellular Carcinoma Through Inhibition of P53", *Cell Physiol Biochem,* Vol. 44 No. 1, pp. 255-266.

Luo, Q., Lin, H., Ye, X., Huang, J., Lu, S. & Xu, L. (2015), "Trim44 facilitates the migration and invasion of human lung cancer cells via the NF-kappaB signaling pathway", *Int J Clin Oncol,* Vol. 20 No. 3, pp. 508-17.

Pan, S., Deng, Y., Fu, J., Zhang, Y., Zhang, Z., Ru, X. & Qin, X. (2019), "TRIM52 promotes colorectal cancer cell proliferation through the STAT3 signaling", *Cancer Cell Int,* Vol. 1957.

Patil, G., Xu, L., Wu, Y., Song, K., Hao, W., Hua, F., Wang, L. & Li, S. (2020), "TRIM41-Mediated Ubiquitination of Nucleoprotein Limits Vesicular Stomatitis Virus Infection", *Viruses,* Vol. 12 No. 2, pp.

Shen, J., Wu, Q., Liang, T., Zhang, J., Bai, J., Yuan, M. & Shen, P. (2021), "TRIM40 inhibits IgA1-induced proliferation of glomerular mesangial cells by inactivating NLRP3 inflammasome through ubiquitination", *Mol Immunol,* Vol. 140225-232.

Shi, G., Lv, C., Yang, Z., Qin, T., Sun, L., Pan, P. & Wang, D. (2019), "TRIM31 promotes proliferation, invasion and migration of glioma cells through Akt signaling pathway", *Neoplasma,* Vol. 66 No. 5, pp. 727-735.

Sparrer, K., Gableske, S., Zurenski, M. A., Parker, Z. M., Full, F., Baumgart, G. J., Kato, J., Pacheco-Rodriguez, G., Liang, C., Pornillos, O., Moss, J., Vaughan, M. & Gack, M. U. (2017), "TRIM23 mediates virus-induced autophagy via activation of TBK1", *Nat Microbiol,* Vol. 2 No. 11, pp. 1543-1557.

Sun, G., Sui, X., Han, D., Gao, J., Liu, Y. & Zhou, L. (2017), "TRIM59 promotes cell proliferation, migration and invasion in human hepatocellular carcinoma cells", *Pharmazie,* Vol. 72 No. 11, pp. 674-679.

Sun, N., Jiang, L., Ye, M., Wang, Y., Wang, G., Wan, X., Zhao, Y., Wen, X., Liang, L., Ma, S., Liu, L., Bu, Z., Chen, H. & Li, C. (2020), "TRIM35 mediates protection against influenza infection by activating TRAF3 and degrading viral PB2", *Protein Cell,* Vol. 11 No. 12, pp. 894-914.

Sun, N., Xue, Y., Dai, T., Li, X. & Zheng, N. (2017), "Tripartite motif containing 25 promotes proliferation and invasion of colorectal cancer cells through TGF-beta signaling", *Biosci Rep,* Vol. 37 No. 4, pp.

Wang, C., Xu, J., Fu, H., Zhang, Y., Zhang, X., Yang, D., Zhu, Z., Wei, Z., Hu, Z., Yan, R. & Cai, Q. (2018), "TRIM32 promotes cell proliferation and invasion by activating beta-catenin signalling in gastric cancer", *J Cell Mol Med,* Vol. 22 No. 10, pp. 5020-5028.

Wang, F., Wang, H., Sun, L., Niu, C. & Xu, J. (2020), "TRIM59 inhibits PPM1A through ubiquitination and activates TGF-beta/Smad signaling to promote the invasion of ectopic endometrial stromal cells in endometriosis", *Am J Physiol Cell Physiol,* Vol. 319 No. 2, pp. C392-C401.

Wang, J., Yang, F., Zhuang, J., Huo, Q., Li, J. & Xie, N. (2022), "TRIM58 inactivates p53/p21 to promote chemoresistance via ubiquitination of DDX3 in breast cancer", *Int J Biochem Cell Biol,* Vol. 143106140.

Wang, K., Zou, C., Wang, X., Huang, C., Feng, T., Pan, W., Wu, Q., Wang, P. & Dai, J. (2018), "Interferon-stimulated TRIM69 interrupts dengue virus replication by ubiquitinating viral nonstructural protein 3", *PLoS Pathog,* Vol. 14 No. 8, pp. e1007287.

Wang, R., Huang, K. L. & Xing, L. X. (2022), "TRIM35 functions as a novel tumor suppressor in breast cancer by inducing cell apoptosis through ubiquitination of PDK1", *Neoplasma,*.

Wang, S., Zhang, Y., Huang, J., Wong, C. C., Zhai, J., Li, C., Wei, G., Zhao, L., Wang, G., Wei, H., Zhao, Z. & Yu, J. (2019), "TRIM67 Activates p53 to Suppress Colorectal Cancer Initiation and Progression", *Cancer Res,* Vol. 79 No. 16, pp. 4086-4098.

Wei, W. S., Chen, X., Guo, L. Y., Li, X. D., Deng, M. H., Yuan, G. J., He, L. Y., Li, Y. H., Zhang, Z. L., Jiang, L. J., Chen, R. X., Ma, X. D., Wei, S., Ma, N. F., Liu, Z. W., Luo, J. H., Zhou, F. J. & Xie, D. (2018), "TRIM65 supports bladder urothelial carcinoma cell aggressiveness by promoting ANXA2 ubiquitination and degradation", *Cancer Lett,* Vol. 43510-22.

Xian, J., Liang, D., Zhao, C., Chen, Y. & Zhu, Q. (2022), "TRIM21 inhibits the osteogenic differentiation of mesenchymal stem cells by facilitating K48 ubiquitination-mediated degradation of Akt", *Exp Cell Res,* Vol. 412 No. 2, pp. 113034.

Xiao, W., Wang, X., Wang, T. & Xing, J. (2018), "TRIM2 downregulation in clear cell renal cell carcinoma affects cell proliferation, migration, and invasion and predicts poor patients' survival", *Cancer Manag Res,* Vol. 105951-5964.

Xie, W., Zhang, Y., Wang, B., Hu, Y., Zhan, B., Wei, F., Tang, J. & Lian, J. (2020), "Tripartite motif containing 24 regulates cell proliferation in colorectal cancer through YAP signaling", *Cancer Med,* Vol. 9 No. 17, pp. 6367-6376.

Xu, G., Guo, Y., Xu, D., Wang, Y., Shen, Y., Wang, F., Lv, Y., Song, F., Jiang, D., Zhang, Y., Lou, Y., Meng, Y., Yang, Y. & Kang, Y. (2017), "TRIM14 regulates cell proliferation and invasion in osteosarcoma via promotion of the AKT signaling pathway", *Sci Rep,* Vol. 742411.

Xu, L., Wu, Q., Zhou, X., Wu, Q. & Fang, M. (2019), "TRIM13 inhibited cell proliferation and induced cell apoptosis by regulating NF-kappaB pathway in non-small-cell lung carcinoma cells", *Gene,* Vol. 715144015.

Yamada, Y., Takayama, K. I., Fujimura, T., Ashikari, D., Obinata, D., Takahashi, S., Ikeda, K., Kakutani, S., Urano, T., Fukuhara, H., Homma, Y. & Inoue, S. (2017), "A novel prognostic factor TRIM44 promotes cell proliferation and migration, and inhibits apoptosis in testicular germ cell tumor", *Cancer Sci,* Vol. 108 No. 1, pp. 32-41.

Yan, J., Li, Q., Mao, A. P., Hu, M. M. & Shu, H. B. (2014), "TRIM4 modulates type I interferon induction and cellular antiviral response by targeting RIG-I for K63-linked ubiquitination", *J Mol Cell Biol,* Vol. 6 No. 2, pp. 154-63.

Yanagi, T., Watanabe, M., Hata, H., Kitamura, S., Imafuku, K., Yanagi, H., Homma, A., Wang, L., Takahashi, H., Shimizu, H. & Hatakeyama, S. (2018), "Loss of TRIM29 Alters Keratin Distribution to Promote Cell Invasion in Squamous Cell Carcinoma", *Cancer Res,* Vol. 78 No. 24, pp. 6795-6806.

Yang, W., Liu, L., Li, C., Luo, N., Chen, R., Li, L., Yu, F. & Cheng, Z. (2018), "TRIM52 plays an oncogenic role in ovarian cancer associated with NF-kB pathway", *Cell Death Dis,* Vol. 9 No. 9, pp. 908.

Zhan, W., Han, T., Zhang, C., Xie, C., Gan, M., Deng, K., Fu, M. & Wang, J. B. (2015), "TRIM59 Promotes the Proliferation and Migration of Non-Small Cell Lung Cancer Cells by Upregulating Cell Cycle Related Proteins", *PLoS One,* Vol. 10 No. 11, pp. e0142596.

Zhang, Y. & Yang, W. B. (2017), "Down-regulation of tripartite motif protein 59 inhibits proliferation, migration and invasion in breast cancer cells", *Biomed Pharmacother,* Vol. 89462-467.

Zhang, Y., Tao, R., Wu, S. S., Xu, C. C., Wang, J. L., Chen, J., Yu, Y. S., Tang, Z. H., Chen, X. H. & Zang, G. Q. (2018), "TRIM52 up-regulation in hepatocellular carcinoma cells promotes proliferation, migration and invasion through the ubiquitination of PPM1A", *J Exp Clin Cancer Res,* Vol. 37 No. 1, pp. 116.

Zhao, Z., Wang, Y., Yun, D., Huang, Q., Meng, D., Li, Q., Zhang, P., Wang, C., Chen, H. & Lu, D. (2020), "TRIM21 overexpression promotes tumor progression by regulating cell proliferation, cell migration and cell senescence in human glioma", *Am J Cancer Res,* Vol. 10 No. 1, pp. 114-130.

Zheng, S., Zhou, C., Wang, Y., Li, H., Sun, Y. & Shen, Z. (2020), "TRIM6 promotes colorectal cancer cells proliferation and response to thiostrepton by TIS21/FoxM1", *J Exp Clin Cancer Res,* Vol. 39 No. 1, pp. 23.

Zhou, C., Zhang, Z., Zhu, X., Qian, G., Zhou, Y., Sun, Y., Yu, W., Wang, J., Lu, H., Lin, F., Shen, Z. & Zheng, S. (2020), "N6-Methyladenosine modification of the TRIM7 positively regulates tumorigenesis and chemoresistance in osteosarcoma through ubiquitination of BRMS1", *EBioMedicine,* Vol. 59102955.

Zhou, L., Deng, Z. Z., Li, H. Y., Jiang, N., Wei, Z. S., Hong, M. F., Chen, X. D., Wang, J. H., Zhang, M. X., Shi, Y. H., Lu, Z. Q. & Huang, X. M. (2019), "TRIM31 promotes glioma proliferation and invasion through activating NF-kappaB pathway", *Onco Targets Ther,* Vol. 122289-2297.

Zurek, B., Schoultz, I., Neerincx, A., Napolitano, L. M., Birkner, K., Bennek, E., Sellge, G., Lerm, M., Meroni, G., Soderholm, J. D. & Kufer, T. A. (2012), "TRIM27 negatively regulates NOD2 by ubiquitination and proteasomal degradation", *PLoS One,* Vol. 7 No. 7, pp. e41255.
